# Foragers of sympatric Asian honey bee species intercept competitor signals by avoiding benzyl acetate from *Apis cerana* alarm pheromone

**DOI:** 10.1038/s41598-017-03806-6

**Published:** 2017-07-27

**Authors:** Ping Wen, Yanan Cheng, Yufeng Qu, Hongxia Zhang, Jianjun Li, Heather Bell, Ken Tan, James Nieh

**Affiliations:** 1 Key Laboratory of Tropical Forest Ecology, Xishuangbanna Tropical Botanical Garden, Chinese Academy of Sciences, Kunming, Yunnan Province 650223 China; 2 Key Laboratory of Economic Plants and Biotechnology, Kunming Institute of Botany, Chinese Academy of Sciences, Kunming, 650201 Yunnan China; 3 Division of Biological Sciences, Section of Ecology, Behavior, and Evolution, University of California, San Diego, La Jolla, California, USA; 4 University of Chinese Academy of Sciences, Beijing, 100049 China

## Abstract

While foraging, animals can form inter- and intraspecific social signalling networks to avoid similar predators. We report here that foragers of different native Asian honey bee species can detect and use a specialized alarm pheromone component, benzyl acetate (BA), to avoid danger. We analysed the volatile alarm pheromone produced by attacked workers of the most abundant native Asian honey bee, *Apis cerana* and tested the responses of other bee species to these alarm signals. As compared to nest guards, *A*. *cerana* foragers produced 3.38 fold higher levels of BA. In foragers, BA and (*E*)-dec-2-en-1-yl acetate (DA) generated the strongest antennal electrophysiological responses. BA was also the only compound that alerted flying foragers and inhibited *A. cerana* foraging. BA thereby decreased *A. cerana* foraging for risky sites. Interestingly, although BA occurs only in trace amounts and is nearly absent in sympatric honeybee species (respectively only 0.07% and 0.44% as much in *A. dorsata* and *A. florea*), these floral generalists detected and avoided BA as strongly as they did to their own alarm pheromone on natural inflorescences. These results demonstrate that competing pollinators can take advantage of alarm signal information provided by other species.

## Introduction

Alarm signalling, defined as one organism using signals to alert another about danger, is widespread and occurs in plants^1^, insects^2^, and vertebrates^3, 4^. Species at the same trophic level can transfer interspecific information about foraging and risk avoidance^5^. By using common information that reliably indicates predation, prey may benefit from shared information^6, 7^. Such information sharing has been demonstrated in tadpoles^8, 9^, fishes^10^, and social insects^2^. This transfer may be beneficial even when the species are competitors. For example, Asian honey bee foragers from different colonies and species can be rivals for limited nectar and pollen resources^11^. Can they use interspecific alarm pheromones for their individual benefit? Although alarm signalling may be individually costly, it can evolve via kin selection^12^ and reciprocal altruism^13^, as exemplified by alarm signals in eusocial organisms. Once such signals have evolved, different colonies of the same species and even different species could benefit by intercepting information about dangerous food locations. Theoretically, they could intercept alarm signal information^14^. As predicted, *A. cerana* can use olfactory eavesdropping to detect and avoid an alarm pheromone component in the sympatric *Apis dorsata* that *A. cerana* does not possess^15^.

Such interception within a species is a by-product of colonies having the same alarm pheromone. However, between species with different alarm pheromone compounds or ratios of these compounds, evolution could favour heterospecific sensitivity to alarm pheromones. Species that often encounter each other, like members of a pollinator guild, could benefit by learning to recognize alarm pheromones produced by heterospecifics. This is similar to the phenomenon of bees avoiding heterospecific “footprint” cuticular hydrocarbon odours that indicate a specific flower has already been visited and therefore is less likely to be rewarding^16^. We hypothesize that different honey bees can learn to associate different honey bee alarm pheromones with danger and thereby reduce their risk of predation during foraging. Such avoidance has implications for pollination, because predators can impose significant non-consumptive effects by causing pollinators to avoid dangerous locations^17^.

In honey bees, alarm pheromones can increase colony fitness by reducing colony recruitment to dangerous locations. For example, *A. mellifera* foragers that detected sting alarm pheromone at a food source significantly reduced their recruitment (less waggle dancing) and increased their production of inhibitory stop signals^18^. Thus, alarm pheromones can also inhibit recruitment communication, providing an olfactory negative feedback signal against the positive feedback signal of the waggle dance.

Honey bee sting alarm pheromones are multi-component blends. Isopentyl acetate (IPA) is the major component of sting alarm pheromone in all honey bee species^19^. The main other previously-reported sting alarm pheromone components (>10% by mass) of each species are benzyl acetate (BA, in *A. mellifera*)^20^, octyl acetate (OA, in *A. mellifera*, *A. cerana*, *A. florea* and *A. dorsata*)^21^, (*E*)-oct-2-en-1-yl acetate (OEA, in *A. mellifera*)^22^, (*E*)-dec-2-en-1-yl acetate (DA, in *A. cerana, A. dorsata*, *A. laboriosa* and *A. florea*)^15, 20, 21, 23^, (*Z*)-eicos-11-en-1-ol (EH, in *A. mellifera* and *A. cerana*)^24, 25^, and *gamma*-octanoic lactone (GOL, in *A. dorsata* and *A. laboriosa*)^26, 27^.

The functions of these different compounds are best understood in the Western honey bee, *A. mellifera*. IPA, OA and BA play a major role in *A. mellifera* nest defence. IPA is most important for initiating an alarm response, but is so volatile that it is less effective at marking the intruder for further attacks^28^. OA is less volatile, and therefore more persistent: it is important for orienting bees towards a moving target^28^. In *A. mellifera*, BA is more effective at increasing the number of fanning workers in the hive, which may be part of a defensive response^28–30^. In *A. mellifera* workers, BA levels also depend upon task specialization^22^. However, the function of BA is otherwise unclear.

Less is known about the effects of different alarm pheromone components in other honey bee species. IPA is the major alarm pheromone in *A. mellifera*, *A. cerana*, *A. dorsata*, and *A. florea*, but natural sting pheromone elicits a longer-lasting reaction than IPA alone^21^. The more persistent DA may provide an orientation cue in *A. dorsata* and *A. florea*, as OA does in *A. mellifera* ^21, 23^. In *A. cerana*, EH is also more persistent than IPA and may provide orientation information^24^.

In addition, components may exert different effects depending upon context. In the context of foraging, bees are not defending their colony but rather fleeing from danger and marking a location as dangerous^26^. For example, GOL and DA are most effective at repelling *A. dorsata* and *A. cerana* foragers, even though GOL is not found in *A. cerana* ^15^. This example of *A. cerana* intercepting an alarm pheromone component of another bee species illustrates the complexity of forager responses to alarm pheromones^15^.

*A. cerana*, *A. dorsata*, and *A. florea* are sympatric tropical Asian honey bee species^11, 15^, face formidable predators at the nest and in the field^15, 19, 31–33^, and are major native pollinators of agricultural crops and native plants in Asia^11, 34–37^. The different species vary in population density, with *A. cerana* as the most common (in order of abundance: *A. cerana* ≥ *A. dorsata* > *A. florea* ^11, 38^). In fact, *A. cerana* is more than three times more abundant than *A. florea* ^38^. The abundance of *A. dorsata* changes seasonally due to their annual migrations. In seasons when *A. dorsata* is sympatric with *A. cerana*, *A. cerana* is initially more abundant than *A. dorsata*, but *A. dorsata* eventually becomes as or more abundant than *A. cerana* ^11, 38^. Thus, it should be advantageous for *A. dorsata* and *A. florea* to detect and intercept the alarm pheromone of *A. cerana*, the most abundant bee species.

Some of these honey bee species have alarm pheromone compounds, like GOL, that are not found in other honey bee species^15^. However, the primary interspecific differences lie in the relative abundances of these different compounds. Because the relative abundances may be a source of information, it is possible that *A. florea* and *A. dorsata* do not respond or respond differently to *A. cerana* alarm pheromone. Our goal was therefore to better understand the function of different honey bee alarm pheromone components in *A. cerana*, to determine if BA varies according to *A. cerana* task specialization, and to test if the sympatric species, *A. dorsata* and *A. florea*, can intercept and use this information.

## Materials and Methods

### Honey bee colonies and sample collection

We used six *A. cerana cerana* colonies (three four-comb colonies and three two-comb observation colonies) at Yunnan Agriculture University and Kunming Botanic Garden in Kunming for the pheromone sampling and feeder experiments. At the Xishuangbanna Tropical Botanical Garden (XTBG), China, approximately 20 *A. cerana* colonies were sited close to where we conducted our inflorescence bioassay experiment. At XTBG, we used naturally foraging bees but conducted trials over several months (October 2015 to April 2016) over a broad area (56 plants distributed over 4 km^2^) and therefore likely used bees from multiple colonies. Sample sizes for each experiment are shown in Table S1.

Wild bee species were collected at XTBG and Nabanhe (Yunnan). *A. dorsata* and *A. florea* foragers were from both XTBG (two colonies per species) and Nabanhe (one colony per species). In field bioassays, we likely used more than three wild colonies of *A. dorsata* because we collected foragers at three different sites, each separated by at least 6.4 km.

### Exp. 1: Alarm pheromone analyses

The headspace odours produced by alarmed *A. dorsata* have been previously determined^15, 26^. To analyse and determine the source of *A. cerana* and *A. florea* alarm pheromone, we collected headspace odours emitted by an alarmed forager using the same procedure used for *A. dorsata* alarm pheromone analyses^15, 26^. Our primary focus was *A. cerana*, but for our information interception experiments, we wished to determine (for *A. florea*) and confirm (for *A. dorsata*), which alarm pheromone components these species possess, using the same procedures with the same Gas Chromatography (GC) and Gas Chromatography-Mass Spectrometry (GC-MS) equipment.

We first carefully captured bees foraging on inflorescences without alarming them. To do so, we gently placed the wider opening of a clean glass funnel (2.0 cm and 0.8 cm diameter openings) over a foraging bee. The captured bee was then induced to walk through the funnel and into a clean 2 ml glass vial, attracted by 365 nm UV LED light (CREE, TW). This vial was sealed with a PTFE cap through which we inserted a needle (0.2 mm in diameter, 5.0 cm long) to disturb the bee into producing alarm pheromone. This needle made contact with, but did not pierce, the bee cuticle. We then removed this needle and collected all volatile alarm pheromones by inserting a 65 μm PDMS/DVB fibre (Supelco, CA) into the vial for Headspace Solid Phase Micro-Extraction (HS-SPME) at 30 °C for 30 min^15^. As a control, in preliminary trials, we captured bees inside glass vials and did not disturb them with a needle. However, even in this situation, we were able to detect trace amounts of known alarm pheromone compounds, such as IPA because the bees were trapped for 30 min. We therefore collected control data on odours pumped from the headspace around undisturbed foragers in a hive and passed through a clean glass tube in which the SPME fibre was placed for 1 hour per trial. We conducted nine trials with three different colonies and found no compounds that matched those produced by needle-alarmed bees.

We also analysed the volatile compounds emitted by sting venom alone. We dissected a sting (stinger and sting gland) from a cold-anesthetized forager or guard and deposited it into a PTFE capped vial (1 sting per vial). After the dissected sting apparatus revived at room temperature, it began to pulse and emit drops of venom. We then extracted the headspace odours using the HS-SPME procedure previously described. For each species, we compared 18 extracts (9 alarmed bees and 9 sting glands from bees from three different *A. cerana* colonies). For *A. dorsata* and *A. florea*, we were not able to collect directly from colonies and therefore collected bees foraging on floral resources at spaced at least 4 km apart.

Our primary focus was on *A. cerana*. To determine if *A. cerana* workers produced different alarm pheromones depending upon their task specialization, we used GC-FID quantification to compare guard bees and forager bees from colonies in Kunming. To collect guard bees, we struck the nest and caught exiting guards with a clean soft cotton sieve. To collect foragers, we captured bees returning to the nest with pollen in their corbiculae with a clean cotton sieve. Such bees are pollen foragers and therefore clearly identifiable as foragers.

For our coupled Gas Chromatography-Electroantennographic Detection (GC-EAD) analyses, we used *A. cerana*, *A. dorsata*, and *A. florea* foragers collected on inflorescences at Kunming and XTBG. EAD couples the measurement of antennal responses with a GC analysis of the compounds from a mixture, natural alarm pheromone. To test antennal responses to known amounts of pure compounds, we used Electroantennography (EAG) with *A. cerana* foragers collected on Kunming inflorescences. Finally, we sampled foragers from colonies for our GC-MS analysis. For our GC and GC-MS chemical analyses and EAG and GC-EAD procedures, we followed standard methods (see Supplemental Methods, S1).

Commercially available isopentyl acetate (IPA), 2-methyl butyl acetate (2MBA), phenyl methanol (PM), octan-1-ol (OH), (*E*)-oct-2-en-1-ol (OEH), isopentyl isopentanoate (IPIP), benzyl acetate (BA), octyl acetate (OA), *gamma*-octanoic lactone (GOL) and phenethyl acetate (PEA) were obtained from TCI Co. Ltd (Tokyo, JP). (*E*)-Dec-2-en-1-ol (DH) was synthesized by NaBH_4_ reduction of (*E*)-dec-2-enal (TCI, JP). (*E*)-Dec-2-en-1-yl acetate (DA) and (*E*)-oct-2-en-1-yl acetate (OEA) were synthesized by acetylation of (*E*)-dec-2-en-1-ol and (*E*)-oct-2-en-1-ol, respectively, using acetylchloride in hexane with triethylamine^39^, and then purified with silica chromatography.

### Exp. 2: *A. cerana* alarm flight experiment

We tested the behaviour of returning foragers responding to alarm pheromone (natural, synthetic mixtures, and synthetic individual components) released at the nest entrance, providing the context of encountering alarm odour in flight. Alarm flight bioassays were conducted on windless sunny days (above 20 °C) in Kunming. To determine which alarm pheromone components would trigger alarm flight, we applied compounds to a clean filter paper strip (15 mm by 4 mm) that was attached to the end of a clean wood stick (2 mm in diameter, 30 cm long) using a clean no. 0 insect pin. After evaporation of the dichloromethane for 10 s (see above), we positioned each filter paper 30 cm away from the hive entrance, at a 45° angle, to avoid blocking the entrance. Bees normally fly in a straight line directly into the colony. Bees responded to the natural release of alarm pheromone by turning away (fly to a track that was in an acute angle (<90 °) with the straight normal returning trail) before entering the nest. We therefore used this turning behaviour as a bioassay of an alerted bee.

To describe this turning behaviour qualitatively, flight motions were recorded with an HDR-CX450 video camera (Sony, Japan) at 50 frames per second (fps), providing a 2.7 × 1.5 m field of view. We randomly selected a subset of returning bees (15 bees from one colony) and digitized their flight tracks with Tracker v4.92 software (Douglas Brown, USA). We digitized the bee’s position each 20 s, beginning when she entered the field of view and ending when she entered nest. These flight tracks only capture a flattened, 2-D perspective, but illustrate the alarm turning behaviour and the looping flights of alarmed bees as compared to the relatively direct entry flights of non-alarmed bees.

We used dichloromethane (DCM) as a solvent for all tested compounds. We tested the following treatments: control (DCM only), natural alarm pheromone (5 sting glands per trial), IPA + OA + BA + DA, BA + DA, IPA, OA, DA, and BA. Treatments were presented in the following order: single compounds, then compounds mixed with BA, and finally compounds without BA. Between treatments, we waited 15 min and ensured that no alerted bees were observed in front of the test colony. We replicated the treatments three times with each colony, each on a separate day.

**Figure 1:**
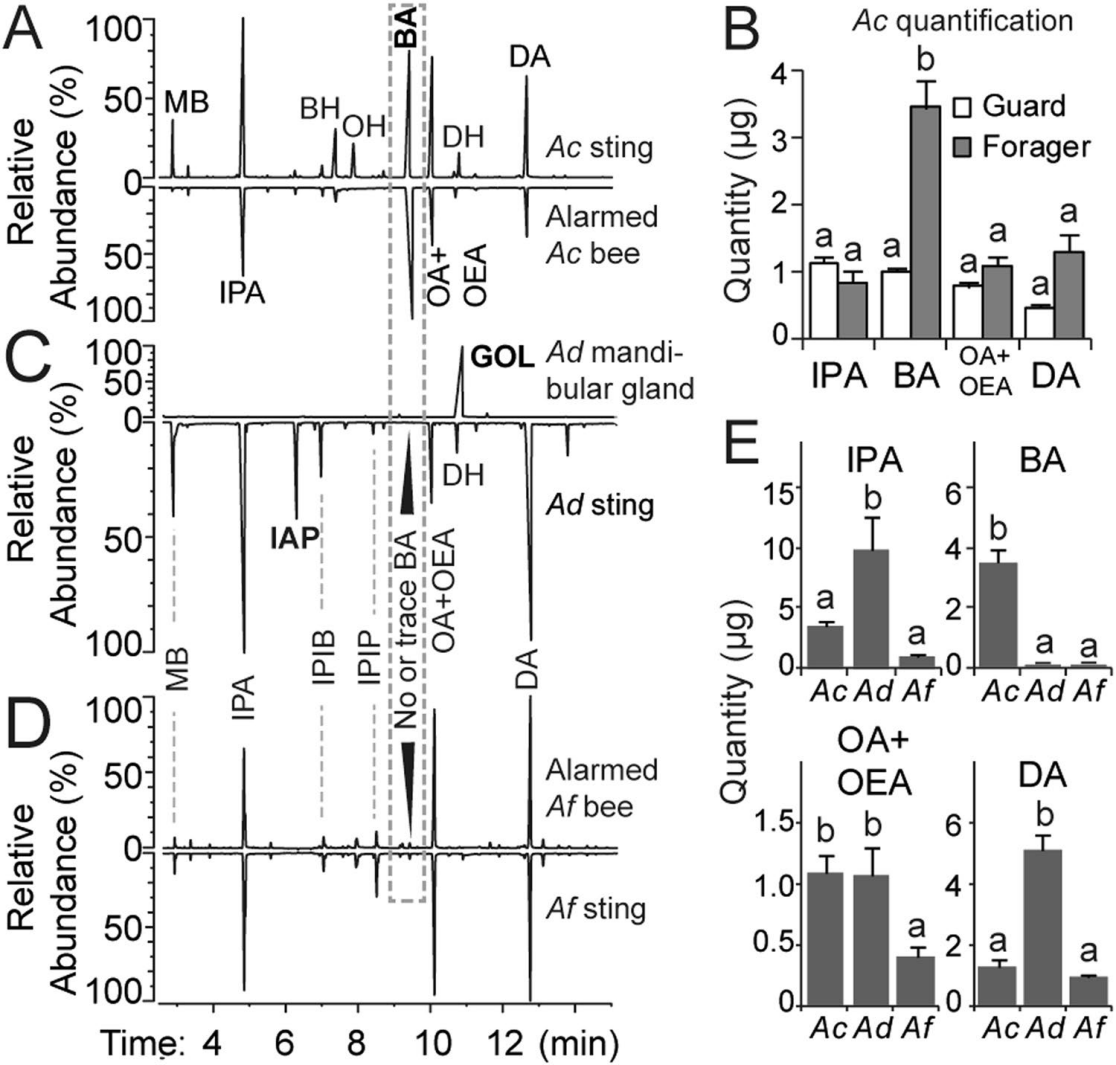
Alarm pheromone components and their relative abundances in three bee species: *A. cerana* (*Ac*), *A. dorsata* (*Ad*), and *A. florea* (*Af*). The following alarm pheromone components are identified: 3-methyl butan-1-ol (MB), isopentyl acetate (IPA), benzyl alcohol (BH), octan-1-ol (OH), isopentyl isobutanoate (IPIB), isopentyl isopentanoate (IPIP), benzyl acetate (BA), octyl acetate (OA), (*E)*-oct-2-en-1-yl acetate (OEA), (*E*)-dec-2-en-1-ol (DH), (*E*)-dec-2-en-1-yl acetate (DA), (*Z*)-11-eicosenol (EH), and *gamma*-octanoic lactone (GOL). (**A**) Typical chromatograms of volatiles produced by the sting gland apparatus of an *Ac* forager (above) and an alarmed *Ac* forager (below). (**B**) Comparison of the quantities of each sting compounds in *Ac* foragers and guards (Tukey’s HSD test, *P* < 0.01). (**C**,**D**) Typical chromatograms of alarm pheromone components in *Ad* (**C**) and (**D**) *Af*. GOL is released by alarmed *Ad* foragers, but is only found in *Ad* mandibular glands^15^. BA is present at such a low trace level in *Ad*, that it is not visible in this overview of the chromatogram. (**E**) Mean quantities of major alarm pheromone compounds found in each of the three species. Bar graphs show means and standard errors. Different letters indicate significant differences based upon Tukey’s HSD tests.

Individual compounds were presented at a quantity of 10 µg/compound, corresponding to about 5 to 10 bee-equivalents (eq), depending upon the compound, Fig. 1B. Mixtures were made of synthetic compounds, with each component added at a 5 bee-equivalent quantity. To determine responses to different quantities of BA, we presented quantities in ascending order: 0 µg control (DCM only), 0.01 μg, 0.1 μg, 1 μg, and 10 μg. To measure the effect of each compound, we counted the first 15 bees that returned to the nest after the test compound or mixture was set out and recorded if each bee exhibited the alarm turning behaviour. Each of trial lasted approximately 1 min.

Given that odours will dissipate over time, we conducted separate trials in which we analysed the number of bees that exhibited alarm behaviour over time, counting the number of alarmed bee each min over 5 min.

### Exp. 3: *A. cerana* feeder foraging alarm experiment

Separately, we tested the behaviour of foragers that encounter alarm pheromone while foraging at a rich sucrose feeder. The most common natural analogue for the experiment would be foragers avoiding alarm pheromone released by a foraging conspecific that had been attacked by a predator. Individually marked foragers were trained to a feeder (an inverted 70 ml glass vial placed on a grooved base) that was 100 m away from the nest, and contained odourless, 35% (w/w) sucrose solution (analytical grade sucrose, AR, Xilongchem, CN) prepared in distilled water. Once the test began, we replaced the training feeder with a clean experimental feeder and, simultaneously, set out an identical control feeder, both displaced by 80 cm from the original training location and spaced 80 cm apart. Identical pieces of filter paper were placed under each feeder. We pipetted out four equidistant dots (2.5 µl each) of IPA, BA, OA, OEA and DA solution onto the filter paper of the experimental feeder and four equidistant dots (2.5 µl each) of DCM solvent on the control feeder. After complete evaporation of the solvent for 10 s, we recorded the choice of each forager. We tested the compounds in ascending quantity order (0.1 µg, 1 µg, 10 µg and 100 µg). Foragers were captured and removed after making a choice, and thus each forager was tested only once. For each sample (four concentrations of five compounds), we recorded the choices of 15 bees from each of three colonies. Each colony was tested on a separate day and under similar sunny weather (15 to 23 °C, moist content 55%).

### Exp. 4: Floral foraging information interception experiments with *A. dorsata* and *A. florea*

Because *A. dorsata* colonies are always wild, typically occur in very tall canopy trees, and exhibit high aggression when approached, it was difficult to train *A. dorsata* to a feeder. Similarly, *A. florea* colonies were difficult to find given that they are not kept for apiculture and occur in the wild in dense brush. For both of these species, we therefore used a floral foraging bioassay to test the response of naturally foraging bees, choosing inflorescences that bees typically visited for longer than 6 s so that we could clearly discern forager responses. We conducted three replicates of Exp. 4 A and Exp. 4B, each on a separate day and under similar weather conditions (sunny, 21 to 30 °C, humidity of 60–75%).

### Exp. 4A: Floral foraging information interception experiments with *A. dorsata*

Both *A. dorsata* and *A. cerana* forage at *Calliandra haematocephala* inflorescences (each inflorescence approximately 3–7 cm in diameter and separated by approximately 10 cm) at XTBG^15^. We therefore used these inflorescences to test if *A. dorsata* could detect and exhibit alarm to the primary components of *A. cerana* alarm pheromone (IPA, GOL, DA, OA, and BA). *A. dorsata* alarm pheromone shares IPA, DA, and OA with *A. cerana* alarm pheromone^15, 26^. However, *A. dorsata* alarm pheromone contains GOL, which *A. cerana* does not^15^. *A. dorsata* forager alarm pheromone does contain trace amounts of BA at 0.07% the level found in an *A. cerana* forager (value based upon GC-FID standard curve shown in Fig. 1E). To deliver odours, we built a stimulus controller (Fig. S4) consisting of a S50-CE air pump (4 ml/s, Nidec, Japan), an active charcoal filter (inner diameter of 1.5 mm), a HXL170 electromagnetic switching gas valve (Zile, China), a PTFE tube, and an odour pipette to deliver the test compounds to an inflorescence visited by honey bees

We only tested one bee at a time. When a bee landed on the inflorescence, we manually triggered a 3 s stimulus of clean, filtered air only (control phase), followed by 1.5 s of the odour stimulus (stimulus phase) added to the airflow. This method ensured that we could control for bee responses to the airflow alone. We recorded if the bee remained or departed during the stimulus phase. Only bees that did not depart during the control phase were used. Overall, only 15.3% of foragers departed in response to the clean airflow only. It was not possible to capture bees since they departed so rapidly in response to alarm pheromone and the same bee could therefore have returned to the same inflorescence, though this was unlikely given the abundance of nearby inflorescences in the *C. haematocephala* tree.

We tested a concentration series of alarm pheromone components deposited on the filter paper strip in the odour pipette. DCM blank controls were tested first, and then the concentrations were tested in ascending order. To avoid odour cross-contamination, the odour paper, odour pipette, and the connecting glass joint (Fig. S4) were replaced with clean ones for each different compound.

### Exp. 4B: Floral foraging information interception experiments with *A. florea* and *A. cerana*

We observed *A. florea* and *A. cerana* foraging together on the large, clustered inflorescences of the date palm, *Phoenix dactylifera*. We conducted nine replicates, three replicates per tree with three different trees. We conducted trials on three separate days between Dec 2015 and Jan 2016. On each day, we conducted three trials, each with a different tree. *A. florea* and *A. cerana* alarm pheromones share IPA, DA, OA, and BA, though *A. florea* alarm pheromone only contains trace amounts of BA (0.44% the quantity found in *A. cerana*, Fig. 1E). In this plant species, the flowers are tightly clustered in a large sheaf (30 cm in diameter, 50 cm in length with multiple small flowers <3 cm apart) and thus targeting the entire inflorescence with the odour pump was not possible. We therefore tested the group response of all bees foraging on a single large inflorescence to odour presented in a paper strip containing DCM as blank or 10 μg (2 to 10 *A. cerana* eq) of IPA, OA, BA, and DA or 5 eq of dissected *A. cerana* or *A. florea* sting glands impregnated on a filter paper strip, respectively, to the inflorescence. DCM was the solvent, and the same volume was used for control and experimental treatments. We waited 30 s to allow the odour to diffuse, and then counted the total number of *A. cerana* and *A. florea* foragers over the next 5 min. The odour was therefore presented for 5.5 min. We waited for approximately 45 min between tests to allow foraging to recover.

### Statistics

For exp. 1A (GC-FID), we analysed component quantities with one-way Analysis of Variance (ANOVA) and used Tukey’s Honestly Significant Difference (HSD) test for post-hoc comparisons. To compare the quantities of BA among the species that we studied, we log-transformed the quantities measured (ng) and used one-way ANOVA, and Tukey’s Honestly Significant Difference (HSD) test for post-hoc comparisons with Sequential-Bonferroni corrected significance levels. We used SPSS 22 (IBM, US) for this analysis.

**Figure 2:**
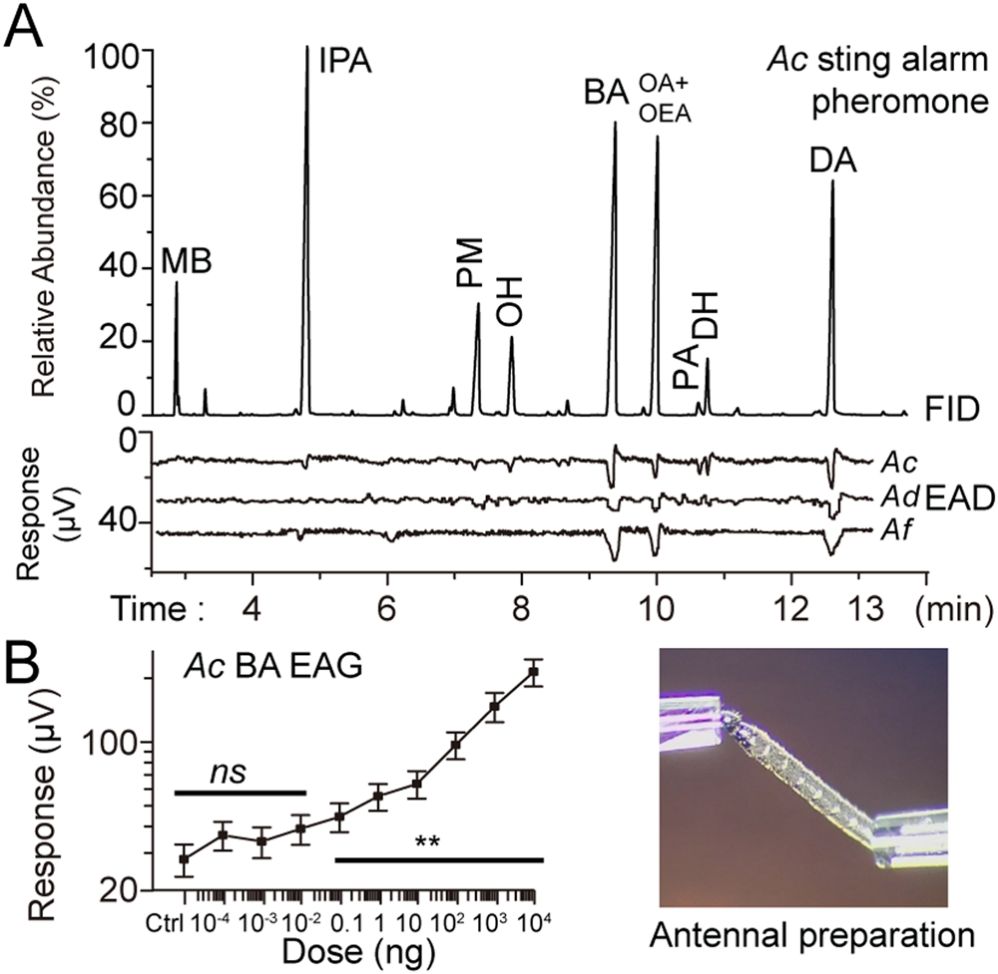
Antennae of foragers from all three bee species (*A. cerana*, *A. dorsata*, and *A. florea*) are highly sensitive to BA. (**A**) GC-EAD responses to the HS-SPME extracts of dissected *A. cerana* stings (1 eq). Relative abundances of each compound and representative EAD signals for each bee species are shown. (**B**) Responses of antennae (EAG) to different quantities of BA EAG (Ctrl = solvent only). The bar (*) indicates quantities that elicited significantly higher responses as compared to the control (Dunnett’s test, *P* < 0.01). Means and standard errors are shown. The inset photo shows the antennal preparation.

For exp. 1B (EAG), we rectified the response data (mV) by subtracting, per bee, the response to each compound from that bee’s response to the blank control and then log-transforming the resulting data. Because bees exhibited no EAG responses to the lowest quantity (0.1 pg, Fig. 2C), we used Dunnett’s method (which corrects for potential Type I error) to make all pairwise comparisons between 0.1 pg and each higher quantity. We included sample sites as a random effect. We used SPSS 22.0 (IBM, US) for this analysis.

For the exp. 2A compound comparison, we used a multiway frequency analysis, based on an ANOVA performed on a generalized linear model (GLM, Poisson model, log link) as described by Vokey^40^ to investigate the effects of different compounds on the number of alarmed bees at the nest entrance. We used a multiway frequency analysis with colony, chemical, and “bee state” (i.e., alarmed or quiet) as categorical factors and number of bees as cell values. For example, one row of data (corresponding to one cell on the 3-dimensional matrix) would have an associated colony number, a chemical, and a binary outcome category variable (e.g., “alarmed”), and the corresponding cell value would then be the number of alarmed bees that were from a given colony when a given chemical was used. We chose this analysis method because it is ideal for repeated-measures discrete data and does not suffer from some of the complexities associated with GLMM. In multiway frequency analysis, all effects, including colony, are treated as fixed. However, similar to specifying factors as random effects in GLMM, this technique allows the model effects to be assessed after the variance due to all other effects is removed (see^40^ for full explanation). For this analysis, we report ﻿*G* ^2^_d﻿f﻿_, the likelihood ratio Chi-square statistic^40^.

For exp. 2B (nest entrance BA quantity-response assay), we analysed the effect of different quantities of BA upon the number of alarmed bees at the nest entrance, also using a multiway frequency analysis with colony, concentration, and bee state as factors and number of bees as cell values.

For exp. 3, we tested if *A. cerana* foragers would avoid feeders with different treatment odors by using Chi-square tests. We used a null hypothesis expectation of equal visitation at both feeders if there was no effect of compound. We used Excel 2007 (Microsoft, US) for this analysis.

For exp. 4A (A. dorsata floral repellence assay), we analysed the A. dorsata data (number of alerted bees) using multiway frequency analysis (R v. 3.3.2). In exp. 4B (A. florea and A. cerana floral repellence assay), we also used a multiway frequency analysis.

Unless otherwise specified, we used JMP 12 (SAS, US) for ANOVA analysis. We present the mean±1 standard error. For our ANOVA, we used residuals analysis to confirm that the data met model assumptions. In the repeated-measures model, we chose a linear model based upon its fit with the data (repeated-measure discrete data). In the experiments that we analysed with ANOVA, each bee was analysed or tested only once to conform to expectations of data independence. For the multiway frequency analysis, the data do not need to conform to a particular distribution or be independent (see^40^ for full discussion).

## Results

### *A. cerana* and *A. florea* alarm odours are produced by the sting glands

In *A. cerana* and *A. florea*, headspace SPME analysis of volatiles emitted by an alarmed forager had the same chemical composition as sting gland volatiles (match for all major peaks, Fig. 1). Thus, all *A. cerana* and *A. florea* alarm odours can be found in the sting gland (Fig. 1A). We therefore proceeded to use only dissected *A. cerana* and *A. florea* sting glands in our subsequent assays testing *A. cerana* and *A. florea* alarm pheromone.

### Exp. 1: BA was more abundant and EAD responsive than other active alarm compounds in *A. cerana* foragers

In *A. cerana*, the sting gland is a major source of alarm pheromone produced by foragers (Fig. 1A). To identify compounds, we used authentic standards and compared the MS spectrum and retention times of these standards, run on the same equipment, with the analyses of natural alarm pheromones. GC-MS analyses showed that sting gland alarm pheromone in guards and foragers consists of the following main components: 3-methyl butanol (MB), isopentyl acetate (IPA), phenyl methanol (PM), (*E*)-oct-2-en-1-ol (OH), benzyl acetate (BA), octyl acetate+(*E*)-Oct-2-en-1-yl acetate (OA+OEA, not distinguishable on the HP-5 column, but are distinct on DB-WAX column, Fig. S1), phenethyl acetate (PA), (*E*)-dec-2-en-1-ol (DH), and (*E*)-dec-2-enyl acetate (DA).

There were significant overall differences in the relative abundance of compounds produced by forager and guard bees (ANOVA: *F*_*7, 112*_ = 22.21, *P* < 0.001, Fig. 1B). Foragers produced significantly more BA than guard bees (Tukey’s HSD test, *P* < *P*_Sequential-Bonferroni_ = 0.01). Among guard bees, all compounds were approximately equally abundant (Tukey’s HSD test, *P* > 0.05, Fig. 1B).

*A. dorsata* and *A. florea* share the same major volatile compounds in their alarm pheromones, including BA (Fig. 1C,D). However, BA is only found trace levels in *A. dorsata* and *A. florea* (Fig. 1E), and probably was not previously identified because it is present at trace levels and may have been confounded with the DA peak.

*A. cerana* forager antennae responded to peaks of IPA, PM, OH, BA, OA+OEA, PA, DH and DA (GC-EAD analysis, Fig. 2A). *A. dorsata* and *A. florea* antennae responded to IPA, BA, OA+OEA and DA.

Because we were primarily interested in *A. cerana*, we focused on this species. *A. cerana* antennae exhibited differential sensitivity (*F*_*8, 72*_ = 73.14, *P* < 0.001). The strongest responses were to BA and DA (Tukey’s HSD: *P* < 0.05, Fig. 2B). As expected, responses increased with higher quantities (EAG quantity within subject effect: *F*_9, 171_ = 432.54, *P* < 0.0001). There was no significant between subject effect of site (*F*_5, 19_ = 0.246, *P* = 0.937 > 0.05) or within subject effect of the interaction site*quantity (*F*_45, 171_ = 1.365, *P* = 0.081 > 0.05).

The difference threshold, the lowest quantity that elicited a significantly greater antennal response than the control, was at 0.1 ng (Dunnett’s method: *P* = 0.002) for BA (Fig. 2C). For comparison, the EAG difference thresholds for IPA, OA and DA were 100 ng, 100 ng and 10 ng, respectively (data from [16] using the same method). Thus, *A*. *cerana* foragers were more sensitive to BA than to any other major alarm pheromone component.

### Exp. 2: BA alarmed flying returning foragers

**Figure 3:**
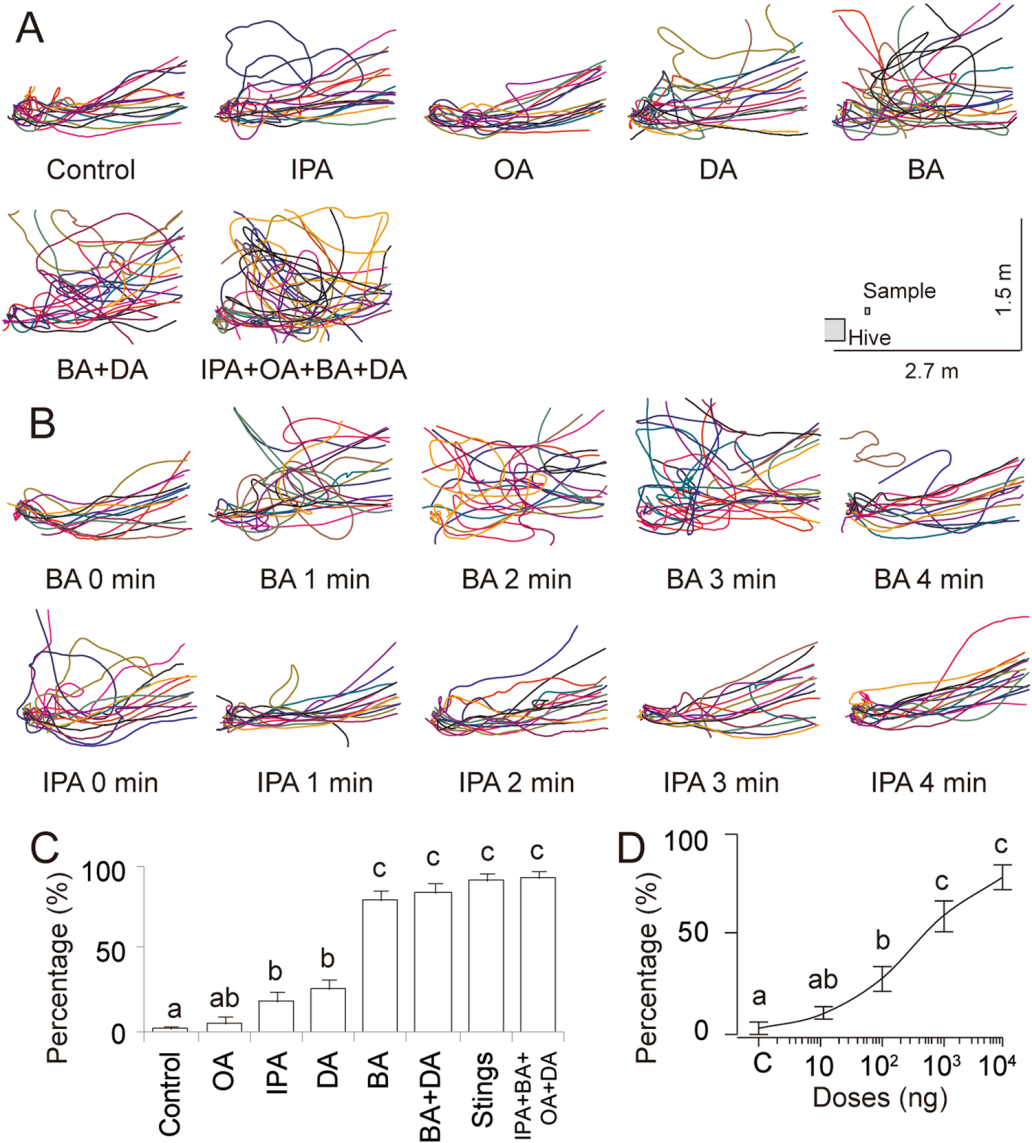
Alarm responses of returning *A. cerana* bees to alarm pheromone components and mixtures in front of the nest. (**A**) Comparison of the flight track of foragers in response to 10 μg (5–10 eq) of the major components separately or in combination. The inset shows a scaled schematic of the hive, sample placement, and video field of view. Bees returning to the nest normally flew directly in (Control). Alarmed bees exhibited sharp turns and looping flights. (**B**) Examples of the effect of time on forager flights in response to BA and IPA. (**C**) Percentage of alarmed bees out of all approaching bees within 6 min in response to different components and mixtures. We presented 10 µg of each component (5–10 eq) or the dissected sting glands from five bees. (**D**) Percentage of alarmed bees to different quantities of BA. Different letters indicate significant differences (Chi-square tests, *P* < 0.01, Sequential Bonferroni corrected). Means and standard errors are shown.

After determining that BA was an abundant alarm pheromone component that elicited strong antennal responses, we tested its efficacy in two different contexts: alarm at the nest entrance (exp. 2) and alarm at food sources (exps. 3&4). We presented the test compounds on paper strips at the nest entrance (Fig. 3A) to simulate sting alarm pheromone release following predator attack or detection. We used a multiway frequency analysis with colony, chemical, and “bee state” (i.e., alarmed or quiet) as categorical factors and number of bees as cell values. So, for example, one row of data (corresponding to one cell on the 3-dimensional matrix) would have an associated colony number, a chemical, and a binary outcome category variable (e.g., “alarmed”), and the corresponding cell value would then be the number of alarmed bees that were present from colony x when chemical x was used. We found a significant effect of compound (*G* ^2^_7_ = 239.14, *P* < 0.0001), but no effect of colony (*G* ^2^_2_ = 3.44, *P* = 0.18), and no interaction between colony and compound (*G* ^2^_14_ = 13.18, *P* = 0.52). Post hoc analyses (Chi-square Z-tests) revealed that BA and mixtures with BA increased alarm responses (*P*≤0.017, Fig. 3C). In addition, BA seemed to act on a different time scale. BA has a lower vapour pressure than IPA and was slower to take effect than IPA (Fig. 3B).

There was a significant effect of compound concentration (*G* ^2^_4_ = 92.74, *P* < 0.0001) (see Fig. 3D), but no significant effect of colony (*G* ^2^_2_ = 0.187, *P* = 0.91) and no colony * concentration interaction (*G* ^2^_8_ = 4.26, *P* = 0.83). The ratios of alerted to observed bees at BA quantities of 0.1, 1﻿ and 10 μg were significantly higher than the ratio of bees responding to the blank control (Chi-square tests: *P* < 0.01 < ﻿*P*_Sequential Bonferroni_ = 0.01). Thus, an alarm response could be elicited by only 0.1 µg of BA, corresponding to 0.03 eq.

### Exp. 3: BA repelled foragers from landing on feeders

**Figure 4:**
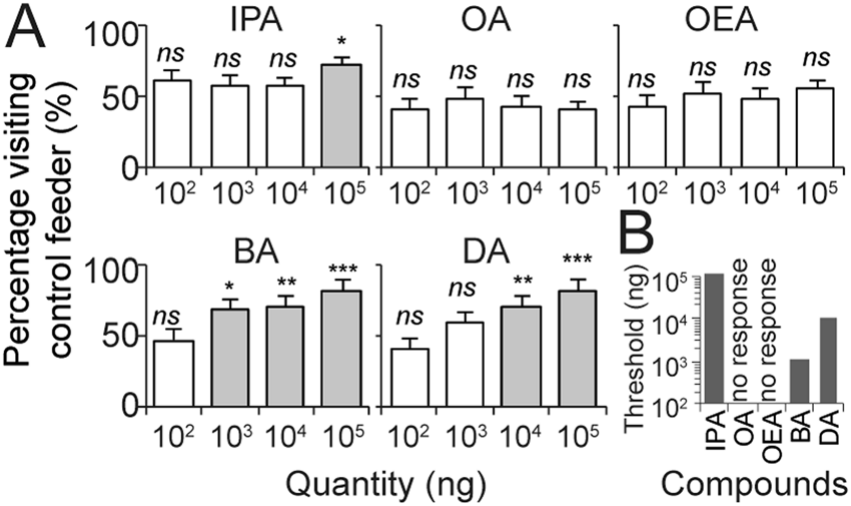
*A. cerana* foragers avoided some *A. cerana* alarm pheromone components in the feeder choice experiment. (**A**) Avoidance of alarm pheromone would result in >50% of bees choosing the control feeder (*P < 0.05, **P < 0.01, ***P < 0.001, *ns* = not significant). (**B**) The difference threshold (the lowest concentration that elicited significant avoidance). Foragers were most sensitive to BA. Means and standard errors are shown.

We next tested the effects of *A. cerana* sting alarm pheromone compounds on bees foraging at a feeder, simulating the situation of a forager encountering alarm pheromone released by an attacked conspecific. Although the feeder offered highly rewarding, concentrated sucrose solution *ad libitum*, IPA, BA, and DA repelled *A. cerana* foragers (Fig. 4A). The minimum repulsive quantities of each compound were: 100 µg IPA (≫1 eq, *χ* ^*2*^_*6*_ = 15.52, n = 45 bees, *P* = 0.017 < 0.050), 1.0 µg of BA (<1 eq, *χ* ^*2*^_*6*_ = 8.49, n = 45, *P* = 0.037 < 0.050, Fig. 4A), and 10 µg of DA (>1 eq, *χ* ^*2*^_*6*_ = 15.52, n = 45, *P* = 0.017 < 0.050). These threshold differences are summarized in Fig. 4B. Thus, foragers were most sensitive to BA, which was the only compound that significantly repelled foragers at < 1 eq.

### Exp. 4: Multiple bee species avoid *A. cerana* alarm pheromones on floral resources

Previously, we found that *A. cerana* could intercept and eavesdrop upon some of the alarm pheromone components of *A. dorsata*, b﻿ut it was unknown if *A. dorsata* or *A. florea* could detect and respond appropriately to *A. cerana* alarm pheromone components.

**Figure 5:**
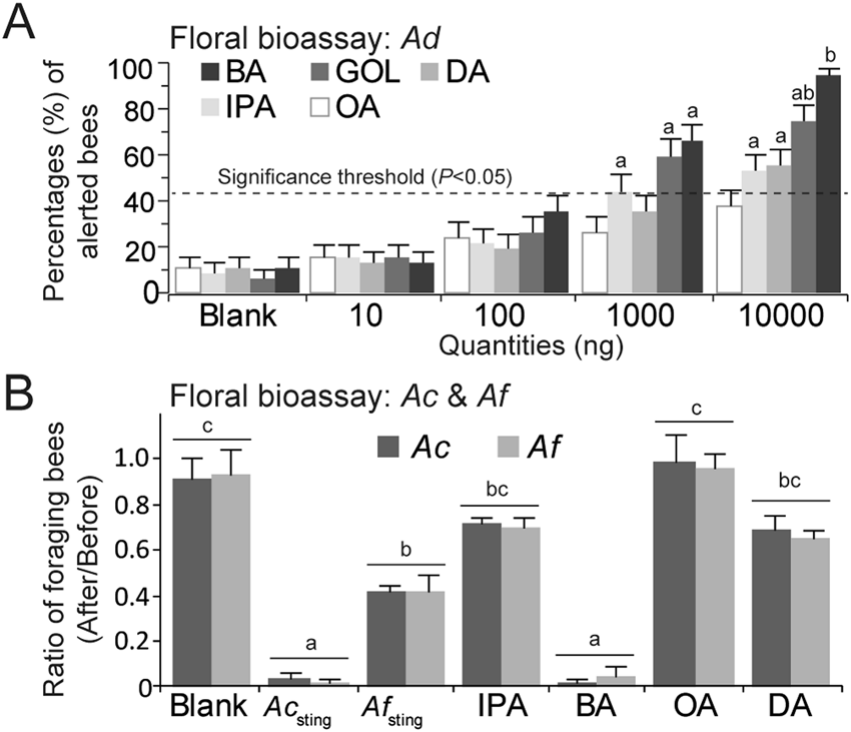
At natural inflorescences, other honey bee species (*A. dorsata* and *A. florea*), can detect and avoid *A. cerana* alarm pheromone components. (**A**) Effect of compound quantity on forager avoidance at *Calliandra haematocephala* inflorescences. Different letters show significant differences as compared to the blank control (*P* < 0.05). (**B**) Effect of different test compounds on the ratio of *A. cerana* (*Ac*) and *A. florea* (*Af* ) foragers choosing to forage at *Phoenix dactylifera* inflorescences after treatment. *Ac*_sting_ and *Af*_sting_ are natural sting gland extracts (1 eq). All other compounds were tested at 5 to 10 *A. cerana* eq. There were no significant differences between *A. cerana* and *A. florea*. If bees avoid the test compound, the ratio < 1. Significant differences between bars or groups of bars are shown with different letters, Tukey’s HSD test, *P* < 0.05). Means and standard errors are shown.

In the floral bioassay of *A. dorsata* (exp. 4A), there were significant effects of compound (*G* ^2^_4_ = 37.35, *P* < 0.0001), concentration (*G* ^2^_5_ = 237.0, *P* < 0.0001) and the compound*quantity interaction (*G* ^2^_16_ = 31.8, *P* = 0.01) Fig. 5). There was no significant effect of field site (*G* ^2^_2_ = 1.3, *P* = 0.52). None of t﻿he other interactions was significant. In pairwise post-hoc comparisons (all tests passed the Sequential Bonferroni correction), the minimum quantities that elicited significant avoidance were: BA at 1 μg (*P* = 0.0000 < 0.001), GOL at 1 μg (*P* = 0.0000 < 0.001), DA at 10 μg (*P* = 0.0001 < 0.001), and IPA at 1 μg (*P* = 0.0065 < 0.01). BA at 10 µg repelled more *A. dorsata* foragers than 1 µg of GOL (*P* = 0.010 < P_Sequential Bonferroni_ = 0.025) or 10 µg of OA (*P* = 0.020 < *P*_Sequential Bonferroni_ = 0.025). However, BA at 10 µg repelled as many foragers as 10 µg of GOL (*P* = 0.10 > 0.05). Thus, A. dorsata was repelled by 0.3 eq of A. cerana BA (Fig. 1E). A. dorsata was also repelled, as expected, by 0.15 eq GOL, an *A. dorsata*-specific alarm component (Fig. 5A).

In the floral bioassay of A. florea and A. cerana (exp. 4B), there were no significant effects of tree (*G* ^2^_2_ = 0.59, *P* = 0.75) or bee species (*G* ^2^_1_ = 0.02, *P* = 0.88), and none of the interactions between any factors was significant (*P* < 0.05). There was a significant effect of compound (*G* ^2^_6_ = 133.67, *P* < 0.0001) such that natural sting alarm pheromones and certain synthetic sting pheromone compounds significantly reduced forager visits. The most inhibitory treatments were *A. cerana* forager natural sting pheromone (5.0 eq) and 10 μg BA (2.8 eq, Fig. 5, Tukey’s HSD, *P* < 0.05).

## Discussion

Signal interception, which can be a form of eavesdropping, has generally been thought of as providing a benefit to the interceptor at the disadvantage of the signaller, although there are exceptions^41^. There are multiple examples of such information usage in stingless bees^42, 43^, ants^44^, termites^45^, social wasps^46^. Here, we considered the case of a pollinator guild in which interspecific sensitivity to a major alarm pheromone component, benzyl acetate (BA), of one of the most common pollinators, *A. cerana*, should also benefit other bee species without, in theory, being detrimental to the signal sender. Such mutualistic or cooperative information sharing occurs in a wide variety of animals, vertebrate and invertebrate^47^. In our case, this mutualism likely arose because many social insects have evolved alarm pheromones that help colonies deal with danger, and there is no evident disadvantage if colonies of the same or different species also use this information to avoid dangerous food or nest predators. In addition, closely related species like the different species of *Apis* share similar alarm signals^15^. One might argue that such information could be used deceptively by one species to deter another from visiting a rich food source. However, the most important selective pressure for the evolution of social insect alarm signals is likely the benefit that alarm signals provide for the colony, the unit of reproduction. It makes little sense for bees to evolve alarm pheromones that deceive competitors but leave their own colonies vulnerable by decreasing the honest information content of alarm signals. Crying wolf is not effective if it reduces ones defences against wolves. We therefore predict that such alarm information will be honestly produced and mutualistically used by sympatric social bees species. Our data support this hypothesis by showing that *A. dorsata, A. florea, and A. cerana* avoided BA on floral food and that *A. cerana* also avoided BA at its own nest.

Our results also support the volatility hypothesis, that some alarm pheromone compounds have been selected based upon their ability to endure and provide lasting information. A potential advantage to intercepting olfactory signals about danger, as compared to typical visual or acoustic danger signals, is that an olfactory danger signal can persist after the signaller departs. However, it may also be important to activate initial defences rapidly. The most volatile compounds, like IPA, can trigger an initial alarm response, but more persistent compounds, such as BA and DA, have greater utility as longer-term markers of danger. The multi-component blends that we find in sting alarm pheromones may therefore have been shaped by their inherent toxicity, detectability, and temporal dynamics (persistence).

For example, we found that *A. cerana* forager sting alarm pheromone is particularly rich in BA, which is significantly less volatile than the most abundant component, IPA. BA has strong effects. At the nest entrance, returning bees, most likely foragers, showed aversive alarm responses to natural sting pheromone, a synthetic combination of four major components, BA, DA, and BA + DA. However, BA stood out as the single compound that most strongly elicited aversion when presented at the nest entrance (Fig. 3C). This behaviour matched the high sensitivity of forager antennae to BA (Fig. 2B).

Although BA has been previously identified in the sting alarm pheromone of *A. mellifera* ^22^, we provide the first identification of BA in *A. cerana*, *A. dorsata* and *A. florea*, likely because the technique previously used, a Carbowax column, coated with solid phase polyethylene glycol (PG) cannot separate DA and BA^21^. Since *A. dorsata* and *A. florea* also produce BA in their respective alarm pheromones, albeit in trace amounts, we cannot state that these two species are eavesdropping upon the BA in *A. cerana* alarm pheromone. Eavesdropping can only be conclusively demonstrated when the eavesdropper does not produce the compound in question^41^. However, we predict that other pollinating bees, particularly non-*Apis* species that are less likely to produce BA, may eavesdrop upon BA to avoid danger.

The higher levels of BA in *A. cerana* foragers as compared to guard sting alarm pheromone matches what is known in *A. mellifera*, in which prior research demonstrated higher levels of BA, BH, and 2-nonanol in foragers than in guards, fanning bees, or comb bees respectively^22^. Elevated levels of BA in foragers may be reasonable, if BA is an important alarm pheromone compound that foragers use to mark dangerous food sources or when encountering danger upon returning to the nest entrance. What is the function of the other alarm pheromone components ? In the context of foraging, prior work showed that (*E*)-dec-2-en-1-yl acetate (DA) was effective at repelling *A. cerana* foragers^15^. OA was identified in all honey bees, but the function of OA in nest defence and repulsing foragers appears to be weak^15, 26, 28–30^. OEA was first identified in *A. mellifera* ^22^, and our analysis also revealed it in *A. cerana*. OEA cannot be separated from OA by using general HP-5 columns^15, 26^, thus OEA may also occur in other *Apis* species and the functions of these two compounds are uncertain. EAD analyses showed that *A. cerana* antennae responded to a peak consisting of OA and OEA (Fig. 2A). It therefore unclear which of these two compounds *A. cerana* can detect. Possibly, the lack of avoidance to OA shown in our assays is due to an inability to detect OA. However, given the relatively high abundance of OA (Fig. S1) and the strong general ability of *A. cerana* to detect multiple volatile compounds within this size range (Fig. 2A), we suggest that *A. cerana* can detect OA.

We hypothesize that the likelihood of encountering BA shapes how useful it is for other bee species. BA is produced in large quantities by *A. cerana*, which also appears to be more common on floral resources than either *A. dorsata* or *A. florea* ^38^ (Table S2). Unfortunately, the exact population densities of *A. dorsata* and *A. florea* are not known. Moreover, *A. dorsata* is a migratory species whose sympatric presence with *A. cerana* can seasonally fluctuate^11^. Thus, testing this hypothesis about species abundance and information utility will require a detailed understanding of species populations in time and space.

### Data accessibility

All data are accessible in supplemental datasheets.

## Electronic supplementary material

Supplementary Information
