# Supplementary material for: Foragers of sympatric Asian honey bee species intercept competitor signals by avoiding benzyl acetate from Apis cerana alarm pheromone
Source: Sci Rep. 2017 Jul 27;7:6721. doi: 10.1038/s41598-017-03806-6 (PMC5532208; doi:10.1038/s41598-017-03806-6)
Supplement: Supplementary file 1 — Supplementary Information [file 41598_2017_3806_MOESM1_ESM.pdf]

Supplementary Information

Foragers of sympatric Asian honey bee species intercept competitor signals by avoiding benzyl acetate from *Apis cerana* alarm pheromone

Ping Wen[1]*, Yanan Cheng[1,4], Yufeng Qu[1], Hongxia Zhang [2], Jianjun Li [1], Heather Bell[3], Ken Tan[1]*, and James Nieh[3]*

*Authors for correspondence

wenping@xtbg.ac.cn

kentan@xtbg.ac.cn

jnieh@ucsd.edu

[1]Key Laboratory of Tropical Forest Ecology, Xishuangbanna Tropical Botanical Garden, Chinese Academy of Sciences, Kunming, Yunnan Province, 650223 China

Tel: 86-871-65227717

Fax: 86-871-65227358

[2]Key Laboratory of Economic Plants and Biotechnology, Kunming Institute of Botany, Chinese Academy of Sciences, Kunming 650201, Yunnan, China

[3]Division of Biological Sciences, Section of Ecology, Behavior, and Evolution, University of California, San Diego, La Jolla, California, USA

[4]University of Chinese Academy of Sciences, Beijing, 100049, China

S1 Supplemental Methods

GC-MS analysis

We followed the same procedure as described in Wang et al 2016. To chemically analyse the alarm odours, we used an HP 7890A-5975C (Agilent, USA) Gas Chromatograph-Mass Spectrometer (GC-MS). An HP-5ms capillary column (30 m × 250 μm × 0.25 μm, Agilent, USA) and a DB-WAX column (30 m × 250 μm × 0.25 μm, Agilent, USA) were used with helium at a flow rate of 37 cm/s as the carrier gas. The oven ramp was set as 50 °C for 2 min, then 5 °C/min to 280 °C for 10 min, when using the HP-5ms column; and the oven ramp was set as 50 °C for 2 min, then 5 °C/min to 230 °C for 10 min when using the DB-WAX column. In the quadrupole mass spectrometry, a 70 eV EI ion source was used at 230 °C. The mass range scanned was m/z 28.5–300 at a rate of 2 × 4 scan/s. We set the abundance detection threshold to 10 in order to detect minor compounds that could easily be fragmented. Data were analysed using Chemstation software (Agilent Technologies, USA). Compounds were verified with external chemical standards.

GC-FID analysis

For quantification of the chemical components, an HP7890B GC was used with a Flame Ionization Detector (FID) and splitless injection at 250 °C. An HP-5 capillary column (30 m × 320 μm × 0.32 μm, Agilent, USA) was used with nitrogen as the carrier gas, flowing at 37 cm/s. The oven ramp was set as 50 °C for 2 min, then 10 °C/min to 280 °C for 5 min. We used external standards to quantify the level of IPA, BA, OA+OEA and DA in each sting gland. To determine the coexistence of OA and OEA, a DB-WAX capillary column (30 m × 320 μm × 0.32 μm, Agilent, USA) was used with oven ramp set as 50 °C for 2 min, then 10 °C/min to 220 °C for 5 min (Figure S1).

GC-EAD and EAG analysis

The antennae of foragers collected on inflorescences at both field sites were used for EAG and GC-EAD analyses. Because bees can specialize on collecting diverse resources (resin, water, salt, pollen, or nectar), we collected foragers on inflorescences to ensure that we studied the antennal responses of floral foragers. We could not be certain of the number of different colonies represented, but these foragers were collected over 1 month from six plots in three different sites: Yunnan Agriculture University (YNAU), Kunming Botanic Garden (KBG), and Xishuangbanna Tropical Botanic Garden (XTBG, 500 km between XTBG and KBG, 4.5 km

between YNAU and KBG). In KBG and XTBG there were at least 32 and 20 colonies, respectively. Thus, these foragers likely came from multiple colonies.

We followed the procedures of Wang et al. [1] for EAG recording. A glass micro-pipette filled with honey bee Ringer's solution was connected to the EAG amplifier input and grounded with Ag/AgCl wires. To obtain the antennae and prevent it from being potentially contaminated with a bee's own alarm pheromone, the bees were drawn into a glass funnel. The smaller funnel end had the same diameter as the bee thorax and head. Bees were lured with a UV light, as described above, head first, into the larger end and then gently and quickly positioned until only the head was exposed from the narrower end. We then randomly severed one antenna (left or right) at the base with iris scissors. The distal end of the antenna was also cut to facilitate recording and the antennae was mounted between the tips of the recording and grounding pipettes (Fig. 2A). The funnel was cleaned after each use.

We presented BA in 1µl dichloromethane (DCM) in the following concentration series (10^{-4}, 10^{-3}, 10^{-2}, 10^{2}, 0.1, 1, 10^{2}, 10^{3}, 10^{4} ng/µl. To evaporate the DCM solvent, we held the paper strip in the open air for 10 s. Preliminary trials with control strips containing DCM showed that bee antennae showed no neural response to DCM after 10 s of such evaporation. Because BA is only moderately volatile (vapour pressure, 0.022 KPa at 25 °C), BA levels were not significantly reduced during this 10 s. We then placed the conditioned filter strip in an odour pipette and immediately used it. We used a new odour pipette for each odour and a new paper strip for each concentration. Each bee was exposed to concentrations in the ascending order.

Improving the sensitivity of EAD recordings is important for enhancing our understanding of insect olfaction [2]. We took the approach of improving upon a classic technique with enhanced instrumentation. We used a custom EAD system coupled to a HP7890B GC. GC conditions were the same as in GC-FID analysis (see above). An HP34465A digital multimeter (Keysight, USA) controlled by BenchVue software (Keysight, USA) running on a PC was used to record the antennal responses. The EAG recording was triggered with the GC trigger signal. The EAG signal was then amplified 21 times by a custom made battery powered amplifier (Fig. S2A).

We custom designed tools for recording from bee antennae. We used micromanipulators (Shengning, CN) with added magnetic bases and glass pipette holders modified from copper drill chucks. A stimulus controller was made with an HXL170 electromagnetic t-valves (Zile, China), a t-joint and timer switches (Yueyu, China), LZB-3 and LZB-4 flow meters (Yuyaozhengxing, China),

two activated charcoal filters (Shuniu, China), and two glass wool humidifiers (Shuniu, China). Teflon tubes or silicon rubber tubes were used to direct the air flows. This EAG system was coupled to the HP7890B GC via a custom made low electromagnetic interference heated transfer line (Fig. S2B).

Electrophysiological recordings demonstrated that *Ac* forager antennae are particularly sensitive to BA and DA (Fig. 2B) and can detect amounts as small as 0.1 and 10 ng, respectively. We were able record responses to such small quantities because of a technical improvement in EAD recordings. There are different ways of improving the sensitivity of EAD, such as using chopper stabilization [2]. We took the approach of improving the basic instrumentation for DC recording and focused on maintaining the original response values by careful amplifier chip selection and instrument design to reduce noise (Fig. S2). Our resulting device was at least 20 to 100 times more sensitive than previous devices (Fig. S3), with a baseline amplitude of 1 to 10 µV. For example, we were able to discriminate minor EAD responses above noise at the very faint 19 µV level.

References

1 Wang, Z. *et al.* Bees eavesdrop upon informative and persistent signal compounds in alarm pheromones. *Sci Rep-Uk* **6**, 25693 (2016).

2 Myrick, A. J. & Baker, T. C. Chopper-stabilized gas chromatography-electroantennography: Part I. Background, signal processing and example. *Biosensors and Bioelectronics* **31**, 197-204 (2012).

S2 Supplemental Table and Figures

Table S1. Sample sizes for each experiment at Yunnan Agriculture University (YNAU), Kunming Botanic Garden (KBG), and Xishuangbanna Tropical Botanic Garden (XTBG).

Experiments	Sites	Plots	Colonies	Number of bees per colony or site (XTBG)	Total # bees
***Ac* GC-MS**	YNAU, KBG	1	3	3 foragers	9
**HS-SPME of *Ac* sting**	YNAU, KBG	1	5	3 guards, 3 foragers	15 guards, 15 foragers
**HS-SPME of *Ac* bee**	YNAU, KBG	1	3	3 foragers	9
***Ac* EAG**	YNAU, KBG, XTBG	6	>6	4, 4, 4, 4, 4, 5 foragers	25
***Ac* GC-EAD**	YNAU, KBG, XTBG	1	1	3 foragers	9
***Ac* alarm flight**	YNAU	1	3	360 for compounds compare, 225 for BA dose-response	585
***Ac* feeder alarm**	YNAU	1	3	15 bees x 5 compounds x 4 concentrations	900
***Ad* GC-MS**	XTBG	3	>3	3 foragers	9
***Af* GC-MS**	XTBG, NBH	3	>3	3 foragers	9
**HS-SPME of *Ad* sting**	XTBG	3	>3	3 foragers	9
**HS-SPME of *Ad* head**	XTBG	3	>3	3 foragers	9
**HS-SPME of *Af* bee**	XTBG, NBH	3	>3	3 foragers	9
**HS-SPME of *Af* sting**	XTBG, NBH	3	>3	3 foragers	9
***Ad* GC-EAD**	XTBG	3	>3	3 foragers	9
***Af* GC-EAD**	XTBG, NBH	3	>3	3 foragers	9
***Ad* on inflorescence alarm**	XTBG	3	>3	15 bees x 5 compounds x 5 concentrations	1125
***Ac* and *Af* on inflorescence alarm**	XTBG	3	>3	*Ac*: 91+109+108 foragers; *Af*: 77+73+83 foragers	*Ac* 308; *Af* 233

Table S2. Data for our hypothesis that that honey bee population abundance may be linked to the use of public information provided by heterospecific sting alarm pheromones by *A. cerana* (*Ac*), *A. dorsata* (*Ad*), and *A. florea* (*Af*). Body length is provided to give a sense of bee size and because body size may be relevant for predators.

**Species**	**Body length**	**Percentage presence in the population of local *Apis* species[1].**	**Signal compound**	**Sources**	**Repellence efficacy[a]**	**Reference**
Ac	*9~13 mm*	40~90%	BA	*Ac*	82%	This study
			GOL	*Ad*	73%	[2]
			DA, IPA	*Ac, Ad, Af*	74%, 70%	This study
Ad	16~17 mm	10~50%	BA	*Ac*	96%	This study
			GOL	*Ad*	76%	[3]
			DA, IPA	*Ac, Ad, Af*	56%, 53%	This study
***Af*[b]**	7~8 mm	0~10%	BA	*Ac*	95%	This study
			DA, IPA	*Ac, Ad, Af*	35%, 30%	This study

[a] Efficacy refers to the maximum level at which bees avoided a feeder or inflorescence with the indicated alarm pheromone compound (feeder tests with *Ac* and inflorescence tests with *Ad* and *Af*).
[b]To measure alarm compound efficacy, we used the reduction in the number of *Af* foragers visiting the treated inflorescence.

References for Table S2

1 Yang, L. & Wu, Y. Species diversity of bees in different habitats in Xishuangbanna tropical forest region. *Chinese Biodiversity* **6**, 8 (1998).
2 Wang, Z. *et al.* Bees eavesdrop upon informative and persistent signal compounds in alarm pheromones. *Sci Rep-Uk* **6**, 25693 (2016).
3 Li, J., Wang, Z., Tan, K., Qu, Y. & Nieh, J. C. Effects of natural and synthetic alarm pheromone and individual pheromone components on foraging behavior of the giant Asian honey bee, *Apis dorsata*. *J Exp Biol* **217**, 3512-3518 (2014).

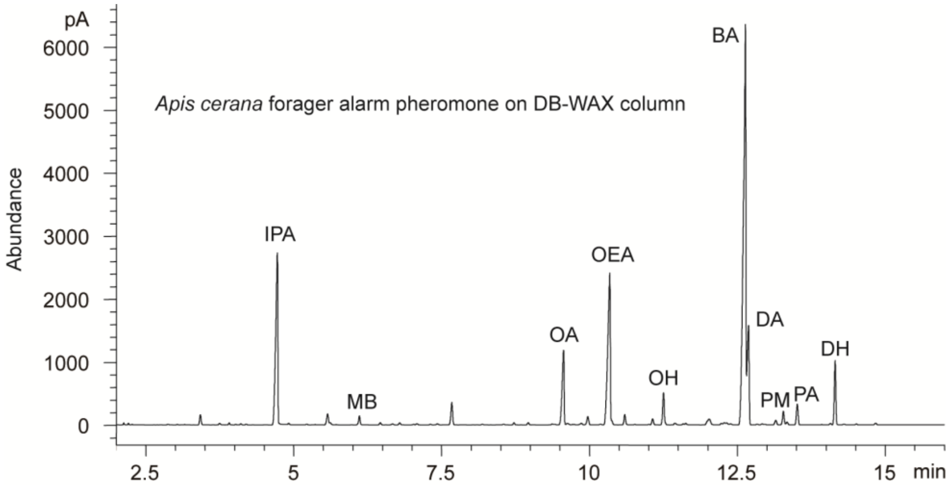

Figure S1. GC profile of *Apis cerana* alarm pheromone on a DB-WAX column. OEA and OA clearly separated on this type of column, but BA and DA was not well distinguished (for comparison see Fig. 3A, which shows the results of using an HP-5m column). We therefore used both types of columns to resolve these different compounds.

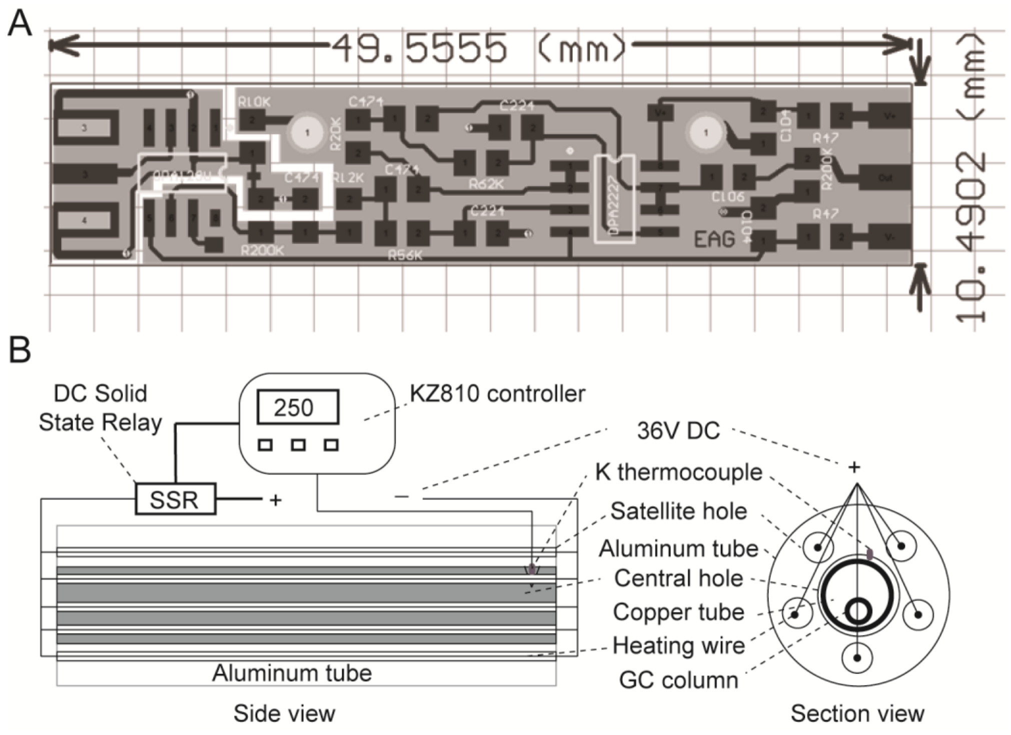

Figure S2. Schematic design of the EAG amplifier and heated transfer line. (A) The board design of the EAG amplifier. The EAG signal was amplified with an OPA 129 UB (TI, USA) high impedance operational amplifier, and then stabilized and filtered with an OPA 2227 (TI, USA) low-noise operational amplifier. To build this amplifier, we selected an OPA 129 chip with highest input impedance out of 20 chips. The input pins of this chip were well-protected on the circuit board to eliminate any leakage currents that could hamper EAG detection. (B) The transfer line consisted of two custom-made 400 mm long aluminium tubes (Dingsheng, CN). The outer tube (8 mm inner diameter, id) had four satellite holes (1 mm id) and a central hole (4.1 mm, id) and contained straight nichrome heating wires (60 W, 20 Ω). The inner tube (3.9 mm outer diameter, od) had a single hole (2.0 mm in diameter), a copper tube (2.0 mm od), a GG-K-30-SLE K type thermocouple (Omega, CH) and a KZ810 temperature controller (Kaize, CN). We used a 36 V DC supply to heat the wires to ensure low levels of electromagnetic interference.

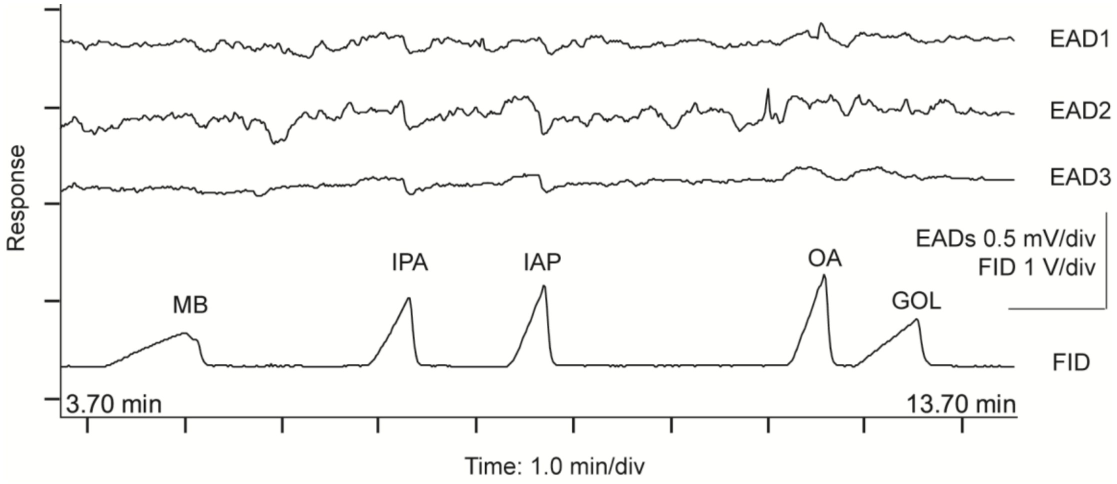

Figure S3. GC-EAD recording of *Apis cerana* antennal responses to synthetic alarm pheromone standards (high up to 10 µg each) using an older commercial instrument (Syntech, combined probe amplifier, IDAC-2 data collector, GC-EAD 2012 software). Responses were hard to discern because of noise. The amplitude of baseline ranged from 0.02 to 0.2 mV.

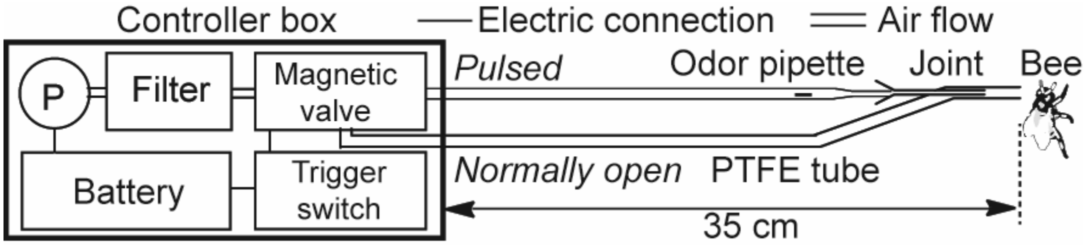

Figure S4. Schematic diagram of the device that provided the odour stimulus in the forager repellence bioassays (P=Pump).

S3 Crude data for statistic figures

Figure 1B FID standard curve quantification

Species	Nest	Extractedbee	Method	Behavior	Beenumber	Compound	Behav*Comp	Quantities(ng)
Ac	1	3	SPME	forager	3	BA	foragerBA	2135
Ac	1	5	SPME	forager	5	BA	foragerBA	3731
Ac	1	6	SPME	forager	6	BA	foragerBA	2474
Ac	2	2	SPME	forager	8	BA	foragerBA	2514
Ac	2	4	SPME	forager	10	BA	foragerBA	1767
Ac	2	5	SPME	forager	11	BA	foragerBA	7205
Ac	3	2	SPME	forager	14	BA	foragerBA	3731
Ac	3	4	SPME	forager	16	BA	foragerBA	5551
Ac	3	5	SPME	forager	17	BA	foragerBA	2971
Ac	4	3	SPME	forager	21	BA	foragerBA	2998
Ac	4	4	SPME	forager	22	BA	foragerBA	3163
Ac	4	5	SPME	forager	23	BA	foragerBA	1978
Ac	5	3	SPME	forager	27	BA	foragerBA	6015
Ac	5	4	SPME	forager	28	BA	foragerBA	3012
Ac	5	6	SPME	forager	30	BA	foragerBA	2527
Ac	1	3	SPME	forager	3	DA	foragerDA	921
Ac	1	5	SPME	forager	5	DA	foragerDA	750
Ac	1	6	SPME	forager	6	DA	foragerDA	253
Ac	2	2	SPME	forager	8	DA	foragerDA	431
Ac	2	4	SPME	forager	10	DA	foragerDA	273
Ac	2	5	SPME	forager	11	DA	foragerDA	3474
Ac	3	2	SPME	forager	14	DA	foragerDA	750
Ac	3	4	SPME	forager	16	DA	foragerDA	1974
Ac	3	5	SPME	forager	17	DA	foragerDA	363
Ac	4	3	SPME	forager	21	DA	foragerDA	2640
Ac	4	4	SPME	forager	22	DA	foragerDA	2684
Ac	4	5	SPME	forager	23	DA	foragerDA	1672
Ac	5	3	SPME	forager	27	DA	foragerDA	1357
Ac	5	4	SPME	forager	28	DA	foragerDA	1170
Ac	5	6	SPME	forager	30	DA	foragerDA	856
Ac	1	3	SPME	forager	3	IPA	foragerIPA	1983
Ac	1	5	SPME	forager	5	IPA	foragerIPA	139
Ac	1	6	SPME	forager	6	IPA	foragerIPA	443
Ac	2	2	SPME	forager	8	IPA	foragerIPA	788
Ac	2	4	SPME	forager	10	IPA	foragerIPA	479
Ac	2	5	SPME	forager	11	IPA	foragerIPA	2118
Ac	3	2	SPME	forager	14	IPA	foragerIPA	139
Ac	3	4	SPME	forager	16	IPA	foragerIPA	116
Ac	3	5	SPME	forager	17	IPA	foragerIPA	374
Ac	4	3	SPME	forager	21	IPA	foragerIPA	367
Ac	4	4	SPME	forager	22	IPA	foragerIPA	1346
Ac	4	5	SPME	forager	23	IPA	foragerIPA	1285
Ac	5	3	SPME	forager	27	IPA	foragerIPA	1787
Ac	5	4	SPME	forager	28	IPA	foragerIPA	429
Ac	5	6	SPME	forager	30	IPA	foragerIPA	951

Ac	1	3	SPME	forager	3	OA	foragerOA	939
Ac	1	5	SPME	forager	5	OA	foragerOA	744
Ac	1	6	SPME	forager	6	OA	foragerOA	529
Ac	2	2	SPME	forager	8	OA	foragerOA	480
Ac	2	4	SPME	forager	10	OA	foragerOA	327
Ac	2	5	SPME	forager	11	OA	foragerOA	1967
Ac	3	2	SPME	forager	14	OA	foragerOA	744
Ac	3	4	SPME	forager	16	OA	foragerOA	1418
Ac	3	5	SPME	forager	17	OA	foragerOA	329
Ac	4	3	SPME	forager	21	OA	foragerOA	1743
Ac	4	4	SPME	forager	22	OA	foragerOA	1638
Ac	4	5	SPME	forager	23	OA	foragerOA	1619
Ac	5	3	SPME	forager	27	OA	foragerOA	1805
Ac	5	4	SPME	forager	28	OA	foragerOA	959
Ac	5	6	SPME	forager	30	OA	foragerOA	1147
Ac	1	1	SPME	Guard	1	BA	GuardBA	951
Ac	1	2	SPME	Guard	2	BA	GuardBA	993
Ac	1	4	SPME	Guard	4	BA	GuardBA	885
Ac	2	1	SPME	Guard	7	BA	GuardBA	1012
Ac	2	3	SPME	Guard	9	BA	GuardBA	1321
Ac	2	6	SPME	Guard	12	BA	GuardBA	749
Ac	3	1	SPME	Guard	13	BA	GuardBA	885
Ac	3	3	SPME	Guard	15	BA	GuardBA	1117
Ac	3	6	SPME	Guard	18	BA	GuardBA	1368
Ac	4	1	SPME	Guard	19	BA	GuardBA	1400
Ac	4	2	SPME	Guard	20	BA	GuardBA	1117
Ac	4	6	SPME	Guard	24	BA	GuardBA	548
Ac	5	1	SPME	Guard	25	BA	GuardBA	856
Ac	5	2	SPME	Guard	26	BA	GuardBA	1169
Ac	5	5	SPME	Guard	29	BA	GuardBA	951
Ac	1	1	SPME	Guard	1	DA	GuardDA	377
Ac	1	2	SPME	Guard	2	DA	GuardDA	131
Ac	1	4	SPME	Guard	4	DA	GuardDA	445
Ac	2	1	SPME	Guard	7	DA	GuardDA	552
Ac	2	3	SPME	Guard	9	DA	GuardDA	489
Ac	2	6	SPME	Guard	12	DA	GuardDA	287
Ac	3	1	SPME	Guard	13	DA	GuardDA	445
Ac	3	3	SPME	Guard	15	DA	GuardDA	331
Ac	3	6	SPME	Guard	18	DA	GuardDA	762
Ac	4	1	SPME	Guard	19	DA	GuardDA	878
Ac	4	2	SPME	Guard	20	DA	GuardDA	331
Ac	4	6	SPME	Guard	24	DA	GuardDA	777
Ac	5	1	SPME	Guard	25	DA	GuardDA	672
Ac	5	2	SPME	Guard	26	DA	GuardDA	239
Ac	5	5	SPME	Guard	29	DA	GuardDA	340
Ac	1	1	SPME	Guard	1	IPA	GuardIPA	1873
Ac	1	2	SPME	Guard	2	IPA	GuardIPA	830
Ac	1	4	SPME	Guard	4	IPA	GuardIPA	1167

Ac	2	1	SPME	Guard	7	IPA	GuardIPA	874
Ac	2	3	SPME	Guard	9	IPA	GuardIPA	1321
Ac	2	6	SPME	Guard	12	IPA	GuardIPA	922
Ac	3	1	SPME	Guard	13	IPA	GuardIPA	1167
Ac	3	3	SPME	Guard	15	IPA	GuardIPA	1042
Ac	3	6	SPME	Guard	18	IPA	GuardIPA	729
Ac	4	1	SPME	Guard	19	IPA	GuardIPA	1257
Ac	4	2	SPME	Guard	20	IPA	GuardIPA	1042
Ac	4	6	SPME	Guard	24	IPA	GuardIPA	993
Ac	5	1	SPME	Guard	25	IPA	GuardIPA	1589
Ac	5	2	SPME	Guard	26	IPA	GuardIPA	1184
Ac	5	5	SPME	Guard	29	IPA	GuardIPA	1156
Ac	1	1	SPME	Guard	1	OA	GuardOA	981
Ac	1	2	SPME	Guard	2	OA	GuardOA	452
Ac	1	4	SPME	Guard	4	OA	GuardOA	585
Ac	2	1	SPME	Guard	7	OA	GuardOA	735
Ac	2	3	SPME	Guard	9	OA	GuardOA	790
Ac	2	6	SPME	Guard	12	OA	GuardOA	1011
Ac	3	1	SPME	Guard	13	OA	GuardOA	585
Ac	3	3	SPME	Guard	15	OA	GuardOA	448
Ac	3	6	SPME	Guard	18	OA	GuardOA	988
Ac	4	1	SPME	Guard	19	OA	GuardOA	990
Ac	4	2	SPME	Guard	20	OA	GuardOA	448
Ac	4	6	SPME	Guard	24	OA	GuardOA	1201
Ac	5	1	SPME	Guard	25	OA	GuardOA	900
Ac	5	2	SPME	Guard	26	OA	GuardOA	633
Ac	5	5	SPME	Guard	29	OA	GuardOA	1098

Figure 1E FID quantification

Apis species compare

BA quantification

Number of bees	Species	amount
1	Ac	2135
2	Ac	3731
3	Ac	2474
4	Ac	2514
5	Ac	1766
6	Ac	7204
7	Ac	3731
8	Ac	5551
9	Ac	2970
10	Ac	2997
11	Ac	3162
12	Ac	1978
13	Ac	6015
14	Ac	3011
15	Ac	2527
1	Ad	5
2	Ad	0
3	Ad	8
4	Ad	0
5	Ad	1
6	Ad	5
7	Ad	3
8	Ad	0
9	Ad	1
1	Af	14
2	Af	23
3	Af	25
4	Af	13
5	Af	10
6	Af	14
7	Af	11
8	Af	24
9	Af	3

IPA quantificaiton

Number of bees	Species	
1	Ac	2135
2	Ac	3731
3	Ac	2474
4	Ac	2514
5	Ac	1766

6	Ac	7204
7	Ac	3731
8	Ac	5551
9	Ac	2970
10	Ac	2997
11	Ac	3162
12	Ac	1978
13	Ac	6015
14	Ac	3011
15	Ac	2527
1	Ad	12554
2	Ad	17245
3	Ad	2006
4	Ad	6993
5	Ad	3312
6	Ad	3297
7	Ad	24546
8	Ad	4087
9	Ad	15158
1	Af	391
2	Af	433
3	Af	696
4	Af	1119
5	Af	2434
6	Af	320
7	Af	1421
8	Af	331
9	Af	1965

DA quantificaiton

Number of bees	Species	
1	Ac	920
2	Ac	749
3	Ac	253
4	Ac	431
5	Ac	273
6	Ac	3474
7	Ac	749
8	Ac	1973
9	Ac	362
10	Ac	2640
11	Ac	2684
12	Ac	1672
13	Ac	1357
14	Ac	1169
15	Ac	855

1	Ad	3247
2	Ad	4031
3	Ad	5072
4	Ad	4974
5	Ad	4860
6	Ad	7500
7	Ad	6534
8	Ad	6306
9	Ad	3973
1	Af	1263
2	Af	355
3	Af	374
4	Af	688
5	Af	1478
6	Af	905
7	Af	1137
8	Af	1235
9	Af	670

OA+OEA quantificaiton

Number of bees	Species	
1	Ac	939
2	Ac	744
3	Ac	529
4	Ac	479
5	Ac	326
6	Ac	1966
7	Ac	744
8	Ac	1417
9	Ac	329
10	Ac	1743
11	Ac	1637
12	Ac	1618
13	Ac	1805
14	Ac	959
15	Ac	1146
1	Ad	563
2	Ad	218
3	Ad	1546
4	Ad	1324
5	Ad	2304
6	Ad	679
7	Ad	234
8	Ad	980
9	Ad	1775
1	Af	171

2	Af	312
3	Af	309
4	Af	180
5	Af	540
6	Af	145
7	Af	877
8	Af	565
9	Af	534

Figure 2A EAD compare

Sites	Bee	Compounc	Response
1	1	MB	0.001
2	2	MB	0.001
3	3	MB	0.001
1	4	MB	0.001
2	5	MB	0.001
3	6	MB	0.001
1	7	MB	0.001
2	8	MB	0.001
3	9	MB	0.001
1	1	IPA	0.005
2	2	IPA	0.005
3	3	IPA	0.006
1	4	IPA	0.005
2	5	IPA	0.005
3	6	IPA	0.004
1	7	IPA	0.005
2	8	IPA	0.006
3	9	IPA	0.010
1	1	PM	0.005
2	2	PM	0.010
3	3	PM	0.010
1	4	PM	0.007
2	5	PM	0.010
3	6	PM	0.004
1	7	PM	0.005
2	8	PM	0.002
3	9	PM	0.010
1	1	OH	0.004
2	2	OH	0.005
3	3	OH	0.006
1	4	OH	0.001
2	5	OH	0.003
3	6	OH	0.004
1	7	OH	0.003
2	8	OH	0.003
3	9	OH	0.006
1	1	BA	0.020
2	2	BA	0.015
3	3	BA	0.021
1	4	BA	0.020
2	5	BA	0.015
3	6	BA	0.018
1	7	BA	0.020
2	8	BA	0.015
3	9	BA	0.030
1	1	OA	0.010

2	2	OA	0.008
3	3	OA	0.015
1	4	OA	0.010
2	5	OA	0.011
3	6	OA	0.010
1	7	OA	0.016
2	8	OA	0.012
3	9	OA	0.015
1	1	PA	0.004
2	2	PA	0.001
3	3	PA	0.001
1	4	PA	0.001
2	5	PA	0.003
3	6	PA	0.002
1	7	PA	0.002
2	8	PA	0.003
3	9	PA	0.003
1	1	DH	0.004
2	2	DH	0.003
3	3	DH	0.002
1	4	DH	0.001
2	5	DH	0.003
3	6	DH	0.004
1	7	DH	0.003
2	8	DH	0.003
3	9	DH	0.002
1	1	DA	0.020
2	2	DA	0.020
3	3	DA	0.020
1	4	DA	0.020
2	5	DA	0.020
3	6	DA	0.015
1	7	DA	0.020
2	8	DA	0.015
3	9	DA	0.025

Figure 2B BA EAG

Sort Order	Exp	Compound	Type	Sites	Plots	Bee	BeeName	Amt tested (ng)	Meter Reads	Response (mV)	LogResponse+2.1	Contrl	Rectified by control
1.000	EAG	BA	synthetic	YNAU	1.000	1.000	11	0.000	1.100	0.052	0.819	0.819	0.000
2.000	EAG	BA	synthetic	YNAU	1.000	1.000	11	0.000	1.200	0.057	0.857	0.819	0.038
3.000	EAG	BA	synthetic	YNAU	1.000	1.000	11	0.001	1.200	0.057	0.857	0.819	0.038
4.000	EAG	BA	synthetic	YNAU	1.000	1.000	11	0.010	1.300	0.062	0.892	0.819	0.073
5.000	EAG	BA	synthetic	YNAU	1.000	1.000	11	0.100	1.600	0.076	0.982	0.819	0.163
6.000	EAG	BA	synthetic	YNAU	1.000	1.000	11	1	2.000	0.095	1.079	0.819	0.260
7.000	EAG	BA	synthetic	YNAU	1.000	1.000	11	10	2.200	0.105	1.120	0.819	0.301
8.000	EAG	BA	synthetic	YNAU	1.000	1.000	11	100	3.000	0.143	1.255	0.819	0.436
9.000	EAG	BA	synthetic	YNAU	1.000	1.000	11	1000	4.500	0.214	1.431	0.819	0.612
10.000	EAG	BA	synthetic	YNAU	1.000	1.000	11	10000	7.000	0.333	1.623	0.819	0.804
11.000	EAG	BA	synthetic	YNAU	1.000	2.000	12	0.000	0.700	0.033	0.623	0.623	0.000
12.000	EAG	BA	synthetic	YNAU	1.000	2.000	12	0.000	1.500	0.071	0.954	0.623	0.331
13.000	EAG	BA	synthetic	YNAU	1.000	2.000	12	0.001	1.500	0.071	0.954	0.623	0.331
14.000	EAG	BA	synthetic	YNAU	1.000	2.000	12	0.010	1.500	0.071	0.954	0.623	0.331
15.000	EAG	BA	synthetic	YNAU	1.000	2.000	12	0.100	1.500	0.071	0.954	0.623	0.331
16.000	EAG	BA	synthetic	YNAU	1.000	2.000	12	1	2.000	0.095	1.079	0.623	0.456
17.000	EAG	BA	synthetic	YNAU	1.000	2.000	12	10	2.100	0.100	1.100	0.623	0.477
18.000	EAG	BA	synthetic	YNAU	1.000	2.000	12	100	3.600	0.171	1.334	0.623	0.711
19.000	EAG	BA	synthetic	YNAU	1.000	2.000	12	1000	5.000	0.238	1.477	0.623	0.854
20.000	EAG	BA	synthetic	YNAU	1.000	2.000	12	10000	8.200	0.390	1.692	0.623	1.069
21.000	EAG	BA	synthetic	YNAU	1.000	3.000	13	0.000	0.300	0.014	0.255	0.255	0.000
22.000	EAG	BA	synthetic	YNAU	1.000	3.000	13	0.000	0.500	0.024	0.477	0.255	0.222
23.000	EAG	BA	synthetic	YNAU	1.000	3.000	13	0.001	0.350	0.017	0.322	0.255	0.067
24.000	EAG	BA	synthetic	YNAU	1.000	3.000	13	0.010	0.430	0.020	0.411	0.255	0.156
25.000	EAG	BA	synthetic	YNAU	1.000	3.000	13	0.100	0.500	0.024	0.477	0.255	0.222
26.000	EAG	BA	synthetic	YNAU	1.000	3.000	13	1	0.700	0.033	0.623	0.255	0.368
27.000	EAG	BA	synthetic	YNAU	1.000	3.000	13	10	0.750	0.036	0.653	0.255	0.398
28.000	EAG	BA	synthetic	YNAU	1.000	3.000	13	100	1.250	0.060	0.875	0.255	0.620
29.000	EAG	BA	synthetic	YNAU	1.000	3.000	13	1000	1.400	0.067	0.924	0.255	0.669
30.000	EAG	BA	synthetic	YNAU	1.000	3.000	13	10000	2.500	0.119	1.176	0.255	0.921
31.000	EAG	BA	synthetic	YNAU	1.000	4.000	14	0.000	0.260	0.012	0.193	0.193	0.000
32.000	EAG	BA	synthetic	YNAU	1.000	4.000	14	0.000	0.400	0.019	0.380	0.193	0.187
33.000	EAG	BA	synthetic	YNAU	1.000	4.000	14	0.001	0.340	0.016	0.309	0.193	0.117
34.000	EAG	BA	synthetic	YNAU	1.000	4.000	14	0.010	0.350	0.017	0.322	0.193	0.129
35.000	EAG	BA	synthetic	YNAU	1.000	4.000	14	0.100	0.450	0.021	0.431	0.193	0.238
36.000	EAG	BA	synthetic	YNAU	1.000	4.000	14	1	0.600	0.029	0.556	0.193	0.363
37.000	EAG	BA	synthetic	YNAU	1.000	4.000	14	10	0.650	0.031	0.591	0.193	0.398
38.000	EAG	BA	synthetic	YNAU	1.000	4.000	14	100	1.060	0.050	0.803	0.193	0.610
39.000	EAG	BA	synthetic	YNAU	1.000	4.000	14	1000	1.300	0.062	0.892	0.193	0.699
40.000	EAG	BA	synthetic	YNAU	1.000	4.000	14	10000	2.200	0.105	1.120	0.193	0.927
41.000	EAG	BA	synthetic	YNAU	2.000	1.000	21	0.000	0.220	0.010	0.120	0.120	0.000
42.000	EAG	BA	synthetic	YNAU	2.000	1.000	21	0.000	0.300	0.014	0.255	0.120	0.135
43.000	EAG	BA	synthetic	YNAU	2.000	1.000	21	0.001	0.250	0.012	0.176	0.120	0.056
44.000	EAG	BA	synthetic	YNAU	2.000	1.000	21	0.010	0.280	0.013	0.225	0.120	0.105
45.000	EAG	BA	synthetic	YNAU	2.000	1.000	21	0.100	0.300	0.014	0.255	0.120	0.135
46.000	EAG	BA	synthetic	YNAU	2.000	1.000	21	1	0.400	0.019	0.380	0.120	0.260
47.000	EAG	BA	synthetic	YNAU	2.000	1.000	21	10	0.430	0.020	0.411	0.120	0.291
48.000	EAG	BA	synthetic	YNAU	2.000	1.000	21	100	0.750	0.036	0.653	0.120	0.533
49.000	EAG	BA	synthetic	YNAU	2.000	1.000	21	1000	0.800	0.038	0.681	0.120	0.561
50.000	EAG	BA	synthetic	YNAU	2.000	1.000	21	10000	1.300	0.062	0.892	0.120	0.772
51.000	EAG	BA	synthetic	YNAU	2.000	2.000	22	0.000	1.700	0.081	1.008	1.008	0.000
52.000	EAG	BA	synthetic	YNAU	2.000	2.000	22	0.000	1.700	0.081	1.008	1.008	0.000
53.000	EAG	BA	synthetic	YNAU	2.000	2.000	22	0.001	1.500	0.071	0.954	1.008	-0.054
54.000	EAG	BA	synthetic	YNAU	2.000	2.000	22	0.010	1.700	0.081	1.008	1.008	0.000
55.000	EAG	BA	synthetic	YNAU	2.000	2.000	22	0.100	2.000	0.095	1.079	1.008	0.071
56.000	EAG	BA	synthetic	YNAU	2.000	2.000	22	1	2.300	0.110	1.140	1.008	0.131
57.000	EAG	BA	synthetic	YNAU	2.000	2.000	22	10	2.800	0.133	1.225	1.008	0.217

58.000	EAG	BA	synthetic	YNAU	2.000	2.000	22	100	4.600	0.219	1.441	1.008	0.432
59.000	EAG	BA	synthetic	YNAU	2.000	2.000	22	1000	4.900	0.233	1.468	1.008	0.460
60.000	EAG	BA	synthetic	YNAU	2.000	2.000	22	10000	12.100	0.576	1.861	1.008	0.852
61.000	EAG	BA	synthetic	YNAU	2.000	3.000	23	0.000	0.360	0.017	0.334	0.334	0.000
62.000	EAG	BA	synthetic	YNAU	2.000	3.000	23	0.000	0.370	0.018	0.346	0.334	0.012
63.000	EAG	BA	synthetic	YNAU	2.000	3.000	23	0.001	0.250	0.012	0.176	0.334	-0.158
64.000	EAG	BA	synthetic	YNAU	2.000	3.000	23	0.010	0.390	0.019	0.369	0.334	0.035
65.000	EAG	BA	synthetic	YNAU	2.000	3.000	23	0.100	0.400	0.019	0.380	0.334	0.046
66.000	EAG	BA	synthetic	YNAU	2.000	3.000	23	1	0.470	0.022	0.450	0.334	0.116
67.000	EAG	BA	synthetic	YNAU	2.000	3.000	23	10	0.500	0.024	0.477	0.334	0.143
68.000	EAG	BA	synthetic	YNAU	2.000	3.000	23	100	0.680	0.032	0.610	0.334	0.276
69.000	EAG	BA	synthetic	YNAU	2.000	3.000	23	1000	0.820	0.039	0.692	0.334	0.358
70.000	EAG	BA	synthetic	YNAU	2.000	3.000	23	10000	1.500	0.071	0.954	0.334	0.620
71.000	EAG	BA	synthetic	YNAU	2.000	4.000	24	0.000	0.280	0.013	0.225	0.225	0.000
72.000	EAG	BA	synthetic	YNAU	2.000	4.000	24	0.000	0.350	0.017	0.322	0.225	0.097
73.000	EAG	BA	synthetic	YNAU	2.000	4.000	24	0.001	0.320	0.015	0.283	0.225	0.058
74.000	EAG	BA	synthetic	YNAU	2.000	4.000	24	0.010	0.340	0.016	0.309	0.225	0.084
75.000	EAG	BA	synthetic	YNAU	2.000	4.000	24	0.100	0.320	0.015	0.283	0.225	0.058
76.000	EAG	BA	synthetic	YNAU	2.000	4.000	24	1	0.500	0.024	0.477	0.225	0.252
77.000	EAG	BA	synthetic	YNAU	2.000	4.000	24	10	0.510	0.024	0.485	0.225	0.260
78.000	EAG	BA	synthetic	YNAU	2.000	4.000	24	100	0.880	0.042	0.722	0.225	0.497
79.000	EAG	BA	synthetic	YNAU	2.000	4.000	24	1000	1.100	0.052	0.819	0.225	0.594
80.000	EAG	BA	synthetic	YNAU	2.000	4.000	24	10000	1.500	0.071	0.954	0.225	0.729
81.000	EAG	BA	synthetic	KBG	3.000	1.000	31	0.000	0.230	0.011	0.140	0.140	0.000
82.000	EAG	BA	synthetic	KBG	3.000	1.000	31	0.000	0.310	0.015	0.269	0.140	0.130
83.000	EAG	BA	synthetic	KBG	3.000	1.000	31	0.001	0.300	0.014	0.255	0.140	0.115
84.000	EAG	BA	synthetic	KBG	3.000	1.000	31	0.010	0.330	0.016	0.296	0.140	0.157
85.000	EAG	BA	synthetic	KBG	3.000	1.000	31	0.100	0.360	0.017	0.334	0.140	0.195
86.000	EAG	BA	synthetic	KBG	3.000	1.000	31	1	0.470	0.022	0.450	0.140	0.310
87.000	EAG	BA	synthetic	KBG	3.000	1.000	31	10	0.510	0.024	0.485	0.140	0.346
88.000	EAG	BA	synthetic	KBG	3.000	1.000	31	100	0.800	0.038	0.681	0.140	0.541
89.000	EAG	BA	synthetic	KBG	3.000	1.000	31	1000	1.150	0.055	0.838	0.140	0.699
90.000	EAG	BA	synthetic	KBG	3.000	1.000	31	10000	1.500	0.071	0.954	0.140	0.814
91.000	EAG	BA	synthetic	KBG	3.000	2.000	32	0.000	0.340	0.016	0.309	0.309	0.000
92.000	EAG	BA	synthetic	KBG	3.000	2.000	32	0.000	0.500	0.024	0.477	0.309	0.167
93.000	EAG	BA	synthetic	KBG	3.000	2.000	32	0.001	0.460	0.022	0.441	0.309	0.131
94.000	EAG	BA	synthetic	KBG	3.000	2.000	32	0.010	0.500	0.024	0.477	0.309	0.167
95.000	EAG	BA	synthetic	KBG	3.000	2.000	32	0.100	0.600	0.029	0.556	0.309	0.247
96.000	EAG	BA	synthetic	KBG	3.000	2.000	32	1	0.920	0.044	0.742	0.309	0.432
97.000	EAG	BA	synthetic	KBG	3.000	2.000	32	10	1.400	0.067	0.924	0.309	0.615
98.000	EAG	BA	synthetic	KBG	3.000	2.000	32	100	2.100	0.100	1.100	0.309	0.791
99.000	EAG	BA	synthetic	KBG	3.000	2.000	32	1000	3.800	0.181	1.358	0.309	1.048
100.000	EAG	BA	synthetic	KBG	3.000	2.000	32	10000	6.100	0.290	1.563	0.309	1.254
101.000	EAG	BA	synthetic	KBG	3.000	3.000	33	0.000	1.900	0.090	1.057	1.057	0.000
102.000	EAG	BA	synthetic	KBG	3.000	3.000	33	0.000	1.900	0.090	1.057	1.057	0.000
103.000	EAG	BA	synthetic	KBG	3.000	3.000	33	0.001	2.300	0.110	1.140	1.057	0.083
104.000	EAG	BA	synthetic	KBG	3.000	3.000	33	0.010	2.800	0.133	1.225	1.057	0.168
105.000	EAG	BA	synthetic	KBG	3.000	3.000	33	0.100	2.600	0.124	1.193	1.057	0.136
106.000	EAG	BA	synthetic	KBG	3.000	3.000	33	1	3.000	0.143	1.255	1.057	0.198
107.000	EAG	BA	synthetic	KBG	3.000	3.000	33	10	2.800	0.133	1.225	1.057	0.168
108.000	EAG	BA	synthetic	KBG	3.000	3.000	33	100	4.500	0.214	1.431	1.057	0.374
109.000	EAG	BA	synthetic	KBG	3.000	3.000	33	1000	7.000	0.333	1.623	1.057	0.566
110.000	EAG	BA	synthetic	KBG	3.000	3.000	33	10000	11.500	0.548	1.838	1.057	0.782
111.000	EAG	BA	synthetic	KBG	3.000	4.000	34	0.000	0.240	0.011	0.158	0.158	0.000
112.000	EAG	BA	synthetic	KBG	3.000	4.000	34	0.000	0.290	0.014	0.240	0.158	0.082
113.000	EAG	BA	synthetic	KBG	3.000	4.000	34	0.001	0.350	0.017	0.322	0.158	0.164
114.000	EAG	BA	synthetic	KBG	3.000	4.000	34	0.010	0.280	0.013	0.225	0.158	0.067
115.000	EAG	BA	synthetic	KBG	3.000	4.000	34	0.100	0.400	0.019	0.380	0.158	0.222
116.000	EAG	BA	synthetic	KBG	3.000	4.000	34	1	0.410	0.020	0.391	0.158	0.233
117.000	EAG	BA	synthetic	KBG	3.000	4.000	34	10	0.570	0.027	0.534	0.158	0.376
118.000	EAG	BA	synthetic	KBG	3.000	4.000	34	100	0.600	0.029	0.556	0.158	0.398

119.000	EAG	BA	synthetic	KBG	3.000	4.000	34	1000	1.220	0.058	0.864	0.158	0.706
120.000	EAG	BA	synthetic	KBG	3.000	4.000	34	10000	2.300	0.110	1.140	0.158	0.982
121.000	EAG	BA	synthetic	KBG	4.000	1.000	41	0.000	0.480	0.023	0.459	0.459	0.000
122.000	EAG	BA	synthetic	KBG	4.000	1.000	41	0.000	0.530	0.025	0.502	0.459	0.043
123.000	EAG	BA	synthetic	KBG	4.000	1.000	41	0.001	0.550	0.026	0.518	0.459	0.059
124.000	EAG	BA	synthetic	KBG	4.000	1.000	41	0.010	0.600	0.029	0.556	0.459	0.097
125.000	EAG	BA	synthetic	KBG	4.000	1.000	41	0.100	0.600	0.029	0.556	0.459	0.097
126.000	EAG	BA	synthetic	KBG	4.000	1.000	41	1	0.900	0.043	0.732	0.459	0.273
127.000	EAG	BA	synthetic	KBG	4.000	1.000	41	10	1.000	0.048	0.778	0.459	0.319
128.000	EAG	BA	synthetic	KBG	4.000	1.000	41	100	1.700	0.081	1.008	0.459	0.549
129.000	EAG	BA	synthetic	KBG	4.000	1.000	41	1000	2.000	0.095	1.079	0.459	0.620
130.000	EAG	BA	synthetic	KBG	4.000	1.000	41	10000	4.000	0.190	1.380	0.459	0.921
131.000	EAG	BA	synthetic	KBG	4.000	2.000	42	0.000	0.400	0.019	0.380	0.380	0.000
132.000	EAG	BA	synthetic	KBG	4.000	2.000	42	0.000	0.520	0.025	0.494	0.380	0.114
133.000	EAG	BA	synthetic	KBG	4.000	2.000	42	0.001	0.410	0.020	0.391	0.380	0.011
134.000	EAG	BA	synthetic	KBG	4.000	2.000	42	0.010	0.600	0.029	0.556	0.380	0.176
135.000	EAG	BA	synthetic	KBG	4.000	2.000	42	0.100	0.680	0.032	0.610	0.380	0.230
136.000	EAG	BA	synthetic	KBG	4.000	2.000	42	1	0.850	0.040	0.707	0.380	0.327
137.000	EAG	BA	synthetic	KBG	4.000	2.000	42	10	1.100	0.052	0.819	0.380	0.439
138.000	EAG	BA	synthetic	KBG	4.000	2.000	42	100	1.600	0.076	0.982	0.380	0.602
139.000	EAG	BA	synthetic	KBG	4.000	2.000	42	1000	1.800	0.086	1.033	0.380	0.653
140.000	EAG	BA	synthetic	KBG	4.000	2.000	42	10000	3.300	0.157	1.296	0.380	0.916
141.000	EAG	BA	synthetic	KBG	4.000	3.000	43	0.000	1.000	0.048	0.778	0.778	0.000
142.000	EAG	BA	synthetic	KBG	4.000	3.000	43	0.000	1.800	0.086	1.033	0.778	0.255
143.000	EAG	BA	synthetic	KBG	4.000	3.000	43	0.001	2.000	0.095	1.079	0.778	0.301
144.000	EAG	BA	synthetic	KBG	4.000	3.000	43	0.010	1.900	0.090	1.057	0.778	0.279
145.000	EAG	BA	synthetic	KBG	4.000	3.000	43	0.100	2.000	0.095	1.079	0.778	0.301
146.000	EAG	BA	synthetic	KBG	4.000	3.000	43	1	2.300	0.110	1.140	0.778	0.362
147.000	EAG	BA	synthetic	KBG	4.000	3.000	43	10	2.600	0.124	1.193	0.778	0.415
148.000	EAG	BA	synthetic	KBG	4.000	3.000	43	100	4.100	0.195	1.391	0.778	0.613
149.000	EAG	BA	synthetic	KBG	4.000	3.000	43	1000	6.100	0.290	1.563	0.778	0.785
150.000	EAG	BA	synthetic	KBG	4.000	3.000	43	10000	11.000	0.524	1.819	0.778	1.041
151.000	EAG	BA	synthetic	KBG	4.000	4.000	44	0.000	0.170	0.008	0.008	0.008	0.000
152.000	EAG	BA	synthetic	KBG	4.000	4.000	44	0.000	0.260	0.012	0.193	0.008	0.185
153.000	EAG	BA	synthetic	KBG	4.000	4.000	44	0.001	0.180	0.009	0.033	0.008	0.025
154.000	EAG	BA	synthetic	KBG	4.000	4.000	44	0.010	0.240	0.011	0.158	0.008	0.150
155.000	EAG	BA	synthetic	KBG	4.000	4.000	44	0.100	0.310	0.015	0.269	0.008	0.261
156.000	EAG	BA	synthetic	KBG	4.000	4.000	44	1	0.400	0.019	0.380	0.008	0.372
157.000	EAG	BA	synthetic	KBG	4.000	4.000	44	10	0.420	0.020	0.401	0.008	0.393
158.000	EAG	BA	synthetic	KBG	4.000	4.000	44	100	0.640	0.030	0.584	0.008	0.576
159.000	EAG	BA	synthetic	KBG	4.000	4.000	44	1000	0.950	0.045	0.756	0.008	0.747
160.000	EAG	BA	synthetic	KBG	4.000	4.000	44	10000	1.750	0.083	1.021	0.008	1.013
161.000	EAG	BA	synthetic	XTBG	5.000	1.000	51	0.000	1.100	0.052	0.819	0.819	0.000
162.000	EAG	BA	synthetic	XTBG	5.000	1.000	51	0.000	1.200	0.057	0.857	0.819	0.038
163.000	EAG	BA	synthetic	XTBG	5.000	1.000	51	0.001	1.200	0.057	0.857	0.819	0.038
164.000	EAG	BA	synthetic	XTBG	5.000	1.000	51	0.010	1.300	0.062	0.892	0.819	0.073
165.000	EAG	BA	synthetic	XTBG	5.000	1.000	51	0.100	1.600	0.076	0.982	0.819	0.163
166.000	EAG	BA	synthetic	XTBG	5.000	1.000	51	1	2.000	0.095	1.079	0.819	0.260
167.000	EAG	BA	synthetic	XTBG	5.000	1.000	51	10	2.200	0.105	1.120	0.819	0.301
168.000	EAG	BA	synthetic	XTBG	5.000	1.000	51	100	3.000	0.143	1.255	0.819	0.436
169.000	EAG	BA	synthetic	XTBG	5.000	1.000	51	1000	4.500	0.214	1.431	0.819	0.612
170.000	EAG	BA	synthetic	XTBG	5.000	1.000	51	10000	7.000	0.333	1.623	0.819	0.804
171.000	EAG	BA	synthetic	XTBG	5.000	2.000	52	0.000	0.270	0.013	0.209	0.209	0.000
172.000	EAG	BA	synthetic	XTBG	5.000	2.000	52	0.000	0.400	0.019	0.380	0.209	0.171
173.000	EAG	BA	synthetic	XTBG	5.000	2.000	52	0.001	0.300	0.014	0.255	0.209	0.046
174.000	EAG	BA	synthetic	XTBG	5.000	2.000	52	0.010	0.380	0.018	0.358	0.209	0.148
175.000	EAG	BA	synthetic	XTBG	5.000	2.000	52	0.100	0.450	0.021	0.431	0.209	0.222
176.000	EAG	BA	synthetic	XTBG	5.000	2.000	52	1	0.600	0.029	0.556	0.209	0.347
177.000	EAG	BA	synthetic	XTBG	5.000	2.000	52	10	0.600	0.029	0.556	0.209	0.347
178.000	EAG	BA	synthetic	XTBG	5.000	2.000	52	100	1.100	0.052	0.819	0.209	0.610
179.000	EAG	BA	synthetic	XTBG	5.000	2.000	52	1000	1.200	0.057	0.857	0.209	0.648

180.000	EAG	BA	synthetic	XTBG	5.000	2.000	52	10000	2.250	0.107	1.130	0.209	0.921
181.000	EAG	BA	synthetic	XTBG	5.000	3.000	53	0.000	0.320	0.015	0.283	0.283	0.000
182.000	EAG	BA	synthetic	XTBG	5.000	3.000	53	0.000	0.480	0.023	0.459	0.283	0.176
183.000	EAG	BA	synthetic	XTBG	5.000	3.000	53	0.001	0.360	0.017	0.334	0.283	0.051
184.000	EAG	BA	synthetic	XTBG	5.000	3.000	53	0.010	0.410	0.020	0.391	0.283	0.108
185.000	EAG	BA	synthetic	XTBG	5.000	3.000	53	0.100	0.550	0.026	0.518	0.283	0.235
186.000	EAG	BA	synthetic	XTBG	5.000	3.000	53	1	0.660	0.031	0.597	0.283	0.314
187.000	EAG	BA	synthetic	XTBG	5.000	3.000	53	10	0.800	0.038	0.681	0.283	0.398
188.000	EAG	BA	synthetic	XTBG	5.000	3.000	53	100	1.300	0.062	0.892	0.283	0.609
189.000	EAG	BA	synthetic	XTBG	5.000	3.000	53	1000	2.000	0.095	1.079	0.283	0.796
190.000	EAG	BA	synthetic	XTBG	5.000	3.000	53	10000	2.800	0.133	1.225	0.283	0.942
191.000	EAG	BA	synthetic	XTBG	5.000	4.000	54	0.000	1.600	0.076	0.982	0.982	0.000
192.000	EAG	BA	synthetic	XTBG	5.000	4.000	54	0.000	2.200	0.105	1.120	0.982	0.138
193.000	EAG	BA	synthetic	XTBG	5.000	4.000	54	0.001	1.400	0.067	0.924	0.982	-0.058
194.000	EAG	BA	synthetic	XTBG	5.000	4.000	54	0.010	1.300	0.062	0.892	0.982	-0.090
195.000	EAG	BA	synthetic	XTBG	5.000	4.000	54	0.100	1.900	0.090	1.057	0.982	0.075
196.000	EAG	BA	synthetic	XTBG	5.000	4.000	54	1	2.200	0.105	1.120	0.982	0.138
197.000	EAG	BA	synthetic	XTBG	5.000	4.000	54	10	2.400	0.114	1.158	0.982	0.176
198.000	EAG	BA	synthetic	XTBG	5.000	4.000	54	100	3.400	0.162	1.309	0.982	0.327
199.000	EAG	BA	synthetic	XTBG	5.000	4.000	54	1000	5.600	0.267	1.526	0.982	0.544
200.000	EAG	BA	synthetic	XTBG	5.000	4.000	54	10000	8.700	0.414	1.717	0.982	0.735
201.000	EAG	BA	synthetic	XTBG	6.000	1.000	61	0.000	0.300	0.014	0.255	0.255	0.000
202.000	EAG	BA	synthetic	XTBG	6.000	1.000	61	0.000	0.400	0.019	0.380	0.255	0.125
203.000	EAG	BA	synthetic	XTBG	6.000	1.000	61	0.001	0.400	0.019	0.380	0.255	0.125
204.000	EAG	BA	synthetic	XTBG	6.000	1.000	61	0.010	0.600	0.029	0.556	0.255	0.301
205.000	EAG	BA	synthetic	XTBG	6.000	1.000	61	0.100	0.440	0.021	0.421	0.255	0.166
206.000	EAG	BA	synthetic	XTBG	6.000	1.000	61	1	0.600	0.029	0.556	0.255	0.301
207.000	EAG	BA	synthetic	XTBG	6.000	1.000	61	10	0.620	0.030	0.570	0.255	0.315
208.000	EAG	BA	synthetic	XTBG	6.000	1.000	61	100	1.000	0.048	0.778	0.255	0.523
209.000	EAG	BA	synthetic	XTBG	6.000	1.000	61	1000	2.100	0.100	1.100	0.255	0.845
210.000	EAG	BA	synthetic	XTBG	6.000	1.000	61	10000	2.800	0.133	1.225	0.255	0.970
211.000	EAG	BA	synthetic	XTBG	6.000	2.000	62	0.000	0.300	0.014	0.255	0.255	0.000
212.000	EAG	BA	synthetic	XTBG	6.000	2.000	62	0.000	0.500	0.024	0.477	0.255	0.222
213.000	EAG	BA	synthetic	XTBG	6.000	2.000	62	0.001	0.500	0.024	0.477	0.255	0.222
214.000	EAG	BA	synthetic	XTBG	6.000	2.000	62	0.010	0.600	0.029	0.556	0.255	0.301
215.000	EAG	BA	synthetic	XTBG	6.000	2.000	62	0.100	0.600	0.029	0.556	0.255	0.301
216.000	EAG	BA	synthetic	XTBG	6.000	2.000	62	1	0.800	0.038	0.681	0.255	0.426
217.000	EAG	BA	synthetic	XTBG	6.000	2.000	62	10	1.200	0.057	0.857	0.255	0.602
218.000	EAG	BA	synthetic	XTBG	6.000	2.000	62	100	1.700	0.081	1.008	0.255	0.753
219.000	EAG	BA	synthetic	XTBG	6.000	2.000	62	1000	3.000	0.143	1.255	0.255	1.000
220.000	EAG	BA	synthetic	XTBG	6.000	2.000	62	10000	5.500	0.262	1.518	0.255	1.263
221.000	EAG	BA	synthetic	XTBG	6.000	3.000	63	0.000	0.350	0.017	0.322	0.322	0.000
222.000	EAG	BA	synthetic	XTBG	6.000	3.000	63	0.000	0.530	0.025	0.502	0.322	0.180
223.000	EAG	BA	synthetic	XTBG	6.000	3.000	63	0.001	0.500	0.024	0.477	0.322	0.155
224.000	EAG	BA	synthetic	XTBG	6.000	3.000	63	0.010	0.680	0.032	0.610	0.322	0.288
225.000	EAG	BA	synthetic	XTBG	6.000	3.000	63	0.100	0.640	0.030	0.584	0.322	0.262
226.000	EAG	BA	synthetic	XTBG	6.000	3.000	63	1	0.870	0.041	0.717	0.322	0.395
227.000	EAG	BA	synthetic	XTBG	6.000	3.000	63	10	1.150	0.055	0.838	0.322	0.517
228.000	EAG	BA	synthetic	XTBG	6.000	3.000	63	100	1.700	0.081	1.008	0.322	0.686
229.000	EAG	BA	synthetic	XTBG	6.000	3.000	63	1000	3.000	0.143	1.255	0.322	0.933
230.000	EAG	BA	synthetic	XTBG	6.000	3.000	63	10000	5.000	0.238	1.477	0.322	1.155
231.000	EAG	BA	synthetic	XTBG	6.000	4.000	64	0.000	0.550	0.026	0.518	0.518	0.000
232.000	EAG	BA	synthetic	XTBG	6.000	4.000	64	0.000	0.550	0.026	0.518	0.518	0.000
233.000	EAG	BA	synthetic	XTBG	6.000	4.000	64	0.001	0.700	0.033	0.623	0.518	0.105
234.000	EAG	BA	synthetic	XTBG	6.000	4.000	64	0.010	1.200	0.057	0.857	0.518	0.339
235.000	EAG	BA	synthetic	XTBG	6.000	4.000	64	0.100	1.900	0.090	1.057	0.518	0.538
236.000	EAG	BA	synthetic	XTBG	6.000	4.000	64	1	2.400	0.114	1.158	0.518	0.640
237.000	EAG	BA	synthetic	XTBG	6.000	4.000	64	10	2.100	0.100	1.100	0.518	0.582
238.000	EAG	BA	synthetic	XTBG	6.000	4.000	64	100	3.000	0.143	1.255	0.518	0.737
239.000	EAG	BA	synthetic	XTBG	6.000	4.000	64	1000	5.300	0.252	1.502	0.518	0.984
240.000	EAG	BA	synthetic	XTBG	6.000	4.000	64	10000	8.600	0.410	1.712	0.518	1.194

241.000	EAG	BA	synthetic	XTBG	6.000	5.000	65	0.000	0.290	0.014	0.240	0.240	0.000
242.000	EAG	BA	synthetic	XTBG	6.000	5.000	65	0.000	0.400	0.019	0.380	0.240	0.140
243.000	EAG	BA	synthetic	XTBG	6.000	5.000	65	0.001	0.320	0.015	0.283	0.240	0.043
244.000	EAG	BA	synthetic	XTBG	6.000	5.000	65	0.010	0.340	0.016	0.309	0.240	0.069
245.000	EAG	BA	synthetic	XTBG	6.000	5.000	65	0.100	0.380	0.018	0.358	0.240	0.117
246.000	EAG	BA	synthetic	XTBG	6.000	5.000	65	1	0.470	0.022	0.450	0.240	0.210
247.000	EAG	BA	synthetic	XTBG	6.000	5.000	65	10	0.440	0.021	0.421	0.240	0.181
248.000	EAG	BA	synthetic	XTBG	6.000	5.000	65	100	0.750	0.036	0.653	0.240	0.413
249.000	EAG	BA	synthetic	XTBG	6.000	5.000	65	1000	0.800	0.038	0.681	0.240	0.441
250.000	EAG	BA	synthetic	XTBG	6.000	5.000	65	10000	1.700	0.081	1.008	0.240	0.768

Figure 3C Compare alarmed bees among selected 15 bees at fixed interval at 10 ug of compounds

NO	Colony	Chemical	Concentra	Alarmed	Non-alarmed	N of selected bees
1	1	BA	10ug	13	2	15
2	2	BA	10ug	12	3	15
3	3	BA	10ug	11	4	15
4	1	BA+DA	10ug+10ug	14	1	15
5	2	BA+DA	10ug+10ug	12	3	15
6	3	BA+DA	10ug+10ug	12	3	15
7	1	Control	0ug	0	15	15
8	2	Control	0ug	1	14	15
9	3	Control	0ug	0	15	15
10	1	DA	10ug	5	10	15
11	2	DA	10ug	4	11	15
12	3	DA	10ug	3	12	15
13	1	Stings	5eq	15	0	15
14	2	Stings	5eq	13	2	15
15	3	Stings	5eq	14	1	15
16	1	IPA	10ug	3	12	15
17	2	IPA	10ug	4	11	15
18	3	IPA	10ug	2	13	15
19	1	IPA+BA+O/	10ug+10ug	14	1	15
20	2	IPA+BA+O/	10ug+10ug	12	3	15
21	3	IPA+BA+O/	10ug+10ug	15	0	15
22	1	OA	10ug	2	13	15
23	2	OA	10ug	1	14	15
24	3	OA	10ug	0	15	15

Figure 3D BA dose-response

NO	Colony	Chemical	Concentra	Alarmed	Non-alarm	N of selected bees
1	1	BA	0	0	15	15
6	2	BA	0	1	14	15
11	3	BA	0	0	15	15
2	1	BA	0.01	1	14	15
7	2	BA	0.01	1	14	15
12	3	BA	0.01	2	13	15
3	1	BA	0.1	4	11	15
8	2	BA	0.1	3	12	15
13	3	BA	0.1	5	10	15
4	1	BA	1	8	7	15
9	2	BA	1	10	5	15
14	3	BA	1	8	7	15
5	1	BA	10	12	3	15
10	2	BA	10	11	4	15
15	3	BA	10	12	3	15

Figure 4A,B Ac feeder

Colony	Chemical	Concentration(ug)	Treated	Control	Total number
1	BA	0.1	7	8	15
2	BA	0.1	9	6	15
3	BA	0.1	8	7	15
1	BA	1	4	11	15
2	BA	1	5	10	15
3	BA	1	5	10	15
1	BA	10	5	10	15
2	BA	10	5	10	15
3	BA	10	3	12	15
1	BA	100	3	12	15
2	BA	100	2	13	15
3	BA	100	3	12	15
1	DA	0.1	9	6	15
2	DA	0.1	9	6	15
3	DA	0.1	8	7	15
1	DA	1	7	8	15
2	DA	1	5	10	15
3	DA	1	6	9	15
1	DA	10	5	10	15
2	DA	10	4	11	15
3	DA	10	4	11	15
1	DA	100	3	12	15
2	DA	100	3	12	15
3	DA	100	2	13	15
1	IPA	0.1	6	9	15
2	IPA	0.1	5	10	15
3	IPA	0.1	7	8	15
1	IPA	1	5	10	15
2	IPA	1	8	7	15
3	IPA	1	6	9	15
1	IPA	10	6	9	15
2	IPA	10	7	8	15
3	IPA	10	6	9	15
1	IPA	100	5	10	15
2	IPA	100	4	11	15
3	IPA	100	4	11	15
1	OA	0.1	8	7	15
2	OA	0.1	9	6	15
3	OA	0.1	10	5	15
1	OA	1	7	8	15
2	OA	1	6	9	15
3	OA	1	10	5	15
1	OA	10	6	9	15
2	OA	10	10	5	15
3	OA	10	10	5	15
1	OA	100	11	4	15

2	OA	100	9	6	15
3	OA	100	7	8	15
1	OEA	0.1	9	6	15
2	OEA	0.1	9	6	15
3	OEA	0.1	8	7	15
1	OEA	1	8	7	15
2	OEA	1	6	9	15
3	OEA	1	8	7	15
1	OEA	10	8	7	15
2	OEA	10	7	8	15
3	OEA	10	8	7	15
1	OEA	100	9	6	15
2	OEA	100	5	10	15
3	OEA	100	6	9	15

Figure 5A Ad on flower

Plant Ch=*Calliandra haematocephala*	Sites	Compound	replication	Concentration	Compound&conc	Number of escaper	Number of tested bees
Ch	1	BA	1	10000ng	BA10000ng	14	15
Ch	2	BA	2	10000ng	BA10000ng	14	15
Ch	3	BA	3	10000ng	BA10000ng	15	15
Ch	1	BA	1	1000ng	BA1000ng	9	15
Ch	2	BA	2	1000ng	BA1000ng	11	15
Ch	3	BA	3	1000ng	BA1000ng	10	15
Ch	1	BA	1	100ng	BA100ng	4	15
Ch	2	BA	2	100ng	BA100ng	6	15
Ch	3	BA	3	100ng	BA100ng	6	15
Ch	1	BA	1	10ng	BA10ng	3	15
Ch	2	BA	2	10ng	BA10ng	3	15
Ch	3	BA	3	10ng	BA10ng	0	15
Ch	1	BA	1	Blank	BABlank	1	15
Ch	2	BA	2	Blank	BABlank	3	15
Ch	3	BA	3	Blank	BABlank	1	15
Ch	1	DA	1	10000ng	DA10000ng	9	15
Ch	2	DA	2	10000ng	DA10000ng	8	15
Ch	3	DA	3	10000ng	DA10000ng	8	15
Ch	1	DA	1	1000ng	DA1000ng	4	15
Ch	2	DA	2	1000ng	DA1000ng	7	15
Ch	3	DA	3	1000ng	DA1000ng	5	15
Ch	1	DA	1	100ng	DA100ng	3	15
Ch	2	DA	2	100ng	DA100ng	4	15
Ch	3	DA	3	100ng	DA100ng	2	15
Ch	1	DA	1	10ng	DA10ng	1	15
Ch	2	DA	2	10ng	DA10ng	3	15
Ch	3	DA	3	10ng	DA10ng	2	15
Ch	1	DA	1	Blank	DABlank	1	15
Ch	2	DA	2	Blank	DABlank	3	15
Ch	3	DA	3	Blank	DABlank	1	15
Ch	1	GOL	1	10000ng	GOL10000ng	11	15
Ch	2	GOL	2	10000ng	GOL10000ng	10	15
Ch	3	GOL	3	10000ng	GOL10000ng	13	15
Ch	1	GOL	1	1000ng	GOL1000ng	7	15
Ch	2	GOL	2	1000ng	GOL1000ng	9	15
Ch	3	GOL	3	1000ng	GOL1000ng	11	15
Ch	1	GOL	1	100ng	GOL100ng	5	15
Ch	2	GOL	2	100ng	GOL100ng	3	15
Ch	3	GOL	3	100ng	GOL100ng	4	15
Ch	1	GOL	1	10ng	GOL10ng	3	15
Ch	2	GOL	2	10ng	GOL10ng	3	15
Ch	3	GOL	3	10ng	GOL10ng	1	15
Ch	1	GOL	1	Blank	GOLBlank	1	15
Ch	2	GOL	2	Blank	GOLBlank	2	15
Ch	3	GOL	3	Blank	GOLBlank	0	15

Ch		GOL+BA	1	100ng+100ng	GOL+BA100ng+100ng	7	15
Ch		GOL+BA	2	100ng+100ng	GOL+BA100ng+100ng	5	15
Ch		GOL+BA	3	100ng+100ng	GOL+BA100ng+100ng	6	15
Ch		GOL+BA	1	500ng+500ng	GOL+BA500ng+500ng	12	15
Ch		GOL+BA	2	500ng+500ng	GOL+BA500ng+500ng	11	15
Ch		GOL+BA	3	500ng+500ng	GOL+BA500ng+500ng	10	15
Ch	1	IPA	1	10000ng	IPA10000ng	5	15
Ch	2	IPA	2	10000ng	IPA10000ng	9	15
Ch	3	IPA	3	10000ng	IPA10000ng	10	15
Ch	1	IPA	1	1000ng	IPA1000ng	6	15
Ch	2	IPA	2	1000ng	IPA1000ng	7	15
Ch	3	IPA	3	1000ng	IPA1000ng	7	15
Ch	1	IPA	1	100ng	IPA100ng	2	15
Ch	2	IPA	2	100ng	IPA100ng	4	15
Ch	3	IPA	3	100ng	IPA100ng	4	15
Ch	1	IPA	1	10ng	IPA10ng	3	15
Ch	2	IPA	2	10ng	IPA10ng	2	15
Ch	3	IPA	3	10ng	IPA10ng	2	15
Ch	1	IPA	1	Blank	IPABlank	1	15
Ch	2	IPA	2	Blank	IPABlank	2	15
Ch	3	IPA	3	Blank	IPABlank	1	15
Ch	1	OA	1	10000ng	OA10000ng	9	15
Ch	2	OA	2	10000ng	OA10000ng	4	15
Ch	3	OA	3	10000ng	OA10000ng	4	15
Ch	1	OA	1	1000ng	OA1000ng	6	15
Ch	2	OA	2	1000ng	OA1000ng	2	15
Ch	3	OA	3	1000ng	OA1000ng	4	15
Ch	1	OA	1	100ng	OA100ng	3	15
Ch	2	OA	2	100ng	OA100ng	4	15
Ch	3	OA	3	100ng	OA100ng	4	15
Ch	1	OA	1	10ng	OA10ng	3	15
Ch	2	OA	2	10ng	OA10ng	3	15
Ch	3	OA	3	10ng	OA10ng	1	15
Ch	1	OA	1	Blank	OABlank	1	15
Ch	2	OA	2	Blank	OABlank	3	15
Ch	3	OA	3	Blank	OABlank	1	15

Figure 5B Ac and Af on flower

Sample s=sting, c=Ac, f=Af — N of forager in 5 min

Field Site	Compound	Compound *Field Site	Amount	Species	Species*compound	# bees before	# bees after	before - after	after/before
1	cs	cs1	5eq	cerana	ceranacs	15	1	14	0.07
2	cs	cs2	5eq	cerana	ceranacs	18	1	17	0.06
3	cs	cs3	5eq	cerana	ceranacs	15	0	15	0.00
1	cs	cs1	5eq	florea	floreacs	11	0	11	0.00
2	cs	cs2	5eq	florea	floreacs	17	1	16	0.06
3	cs	cs3	5eq	florea	floreacs	13	0	13	0.00
1	fs	fs1	5eq	cerana	ceranafs	15	6	9	0.40
2	fs	fs2	5eq	cerana	ceranafs	13	5	8	0.38
3	fs	fs3	5eq	cerana	ceranafs	21	10	11	0.48
1	fs	fs1	5eq	florea	floreafs	9	5	4	0.56
2	fs	fs2	5eq	florea	floreafs	11	3	8	0.27
3	fs	fs3	5eq	florea	floreafs	12	5	7	0.42
1	BA	BA1	10ug	cerana	ceranaBA	13	0	13	0.00
2	BA	BA2	10ug	cerana	ceranaBA	17	1	16	0.06
3	BA	BA3	10ug	cerana	ceranaBA	12	0	12	0.00
1	BA	BA1	10ug	florea	floreaBA	12	0	12	0.00
2	BA	BA2	10ug	florea	floreaBA	7	1	6	0.14
3	BA	BA3	10ug	florea	floreaBA	10	0	10	0.00
1	CK	CK1	10ug	cerana	ceranaCK	11	10	1	0.91
2	CK	CK2	10ug	cerana	ceranaCK	12	13	-1	1.08
3	CK	CK3	10ug	cerana	ceranaCK	16	12	4	0.75
1	CK	CK1	10ug	florea	floreaCK	12	9	3	0.75
2	CK	CK2	10ug	florea	floreaCK	9	8	1	0.89
3	CK	CK3	10ug	florea	floreaCK	13	15	-2	1.15
1	DA	DA1	10ug	cerana	ceranaDA	12	9	3	0.75
2	DA	DA2	10ug	cerana	ceranaDA	17	13	4	0.76
3	DA	DA3	10ug	cerana	ceranaDA	9	5	4	0.56
1	DA	DA1	10ug	florea	floreaDA	12	7	5	0.58
2	DA	DA2	10ug	florea	floreaDA	7	5	2	0.71
3	DA	DA3	10ug	florea	floreaDA	6	4	2	0.67
1	IPA	IPA1	10ug	cerana	ceranaIPA	12	8	4	0.67
2	IPA	IPA2	10ug	cerana	ceranaIPA	16	12	4	0.75
3	IPA	IPA3	10ug	cerana	ceranaIPA	20	15	5	0.75
1	IPA	IPA1	10ug	florea	floreaIPA	8	5	3	0.63
2	IPA	IPA2	10ug	florea	floreaIPA	10	8	2	0.80
3	IPA	IPA3	10ug	florea	floreaIPA	12	8	4	0.67
1	OA	OA1	10ug	cerana	ceranaOA	13	10	3	0.77
2	OA	OA2	10ug	cerana	ceranaOA	16	16	0	1.00
3	OA	OA3	10ug	cerana	ceranaOA	15	18	-3	1.20
1	OA	OA1	10ug	florea	floreaOA	13	12	1	0.92

2	OA	OA2	10ug	florea	floreaOA	12	13	-1	1.08
3	OA	OA3	10ug	florea	floreaOA	17	15	2	0.88
